# Review: Filament Winding and Automated Fiber Placement with In Situ Consolidation for Fiber Reinforced Thermoplastic Polymer Composites

**DOI:** 10.3390/polym13121951

**Published:** 2021-06-11

**Authors:** Yi Di Boon, Sunil Chandrakant Joshi, Somen Kumar Bhudolia

**Affiliations:** School of Mechanical and Aerospace Engineering, Nanyang Technological University, 50 Nanyang Avenue, Singapore 639798, Singapore; mscjoshi@ntu.edu.sg

**Keywords:** thermoplastic resin, process modeling, filament winding, automated fiber placement

## Abstract

Fiber reinforced thermoplastic composites are gaining popularity in many industries due to their short consolidation cycles, among other advantages over thermoset-based composites. Computer aided manufacturing processes, such as filament winding and automated fiber placement, have been used conventionally for thermoset-based composites. The automated processes can be adapted to include in situ consolidation for the fabrication of thermoplastic-based composites. In this paper, a detailed literature review on the factors affecting the in situ consolidation process is presented. The models used to study the various aspects of the in situ consolidation process are discussed. The processing parameters that gave good consolidation results in past studies are compiled and highlighted. The parameters can be used as reference points for future studies to further improve the automated manufacturing processes.

## 1. Introduction

Fiber reinforced polymer (FRP) composites have very high specific strength and stiffness. These favorable properties led to FRP composites becoming more popular in many industries, such as aerospace, automotive, sports, construction, offshore, and so on [1,2,3,4,5,6,7]. Advancements in computer aided manufacturing processes for FRP composites, such as automated fiber placement (AFP) and filament winding (FW), have greatly improved the production throughput and the quality of FRP composite components. Epoxy, a thermoset polymer, is commonly used as the matrix material in FRP composites because of its excellent mechanical strength. However, one of the major drawbacks of thermoset based FRP composites is their long curing cycle, which results in increased manufacturing costs. In contrast, the manufacturing of fiber reinforced thermoplastic polymer (FRTP) composites is generally much less time consuming due to their short consolidation cycles. Besides this, FRTP composites also have other advantages over thermoset based FRP composites, including higher toughness, long shelf life, ease of repairing and potential for recycling [4]. High performance thermoplastic polymers, such as polyether ether ketone (PEEK), can also maintain their mechanical properties in environments with elevated temperature and moisture [8,9]. For FRTP composite components, their fabrication using AFP and FW processes can include in situ consolidation (ISC) to further shorten the processing time required.

Many studies have been carried out on the manufacturing of FRTP composites using AFP and FW with ISC. Researchers used various material forms and ISC methods in their studies, leading to different heat transfer and consolidation mechanisms. In this review, a comprehensive overview on the topic, encompassing the different material forms and ISC methods, is presented. Experimental and numerical studies on the factors affecting the ISC process are discussed, with attention given to recent studies. The optimization of the AFP and FW processes with ISC is deliberated, with emphasis given to the physical and mechanical properties of the FRTP component fabricated, as well as the productivity of the process. A summary of the optimized processing parameters from past studies is also presented, providing a basis for future studies on further improving the fabrication of FRTP components using AFP and FW with ISC.

## 2. Manufacturing of FRTP Composites

The manufacturing of FRTP components generally involves three stages, namely heating, consolidation and cooling. In the heating stage, the thermoplastic matrix is melted or softened so that bonding can occur. Pressure is applied in the consolidation stage to produce components with low void content. During the cooling stage, the cooling rate is controlled to obtain the desired microstructure in the thermoplastic [4].

The autoclave process and compression molding are two commonly used manufacturing processes for FRTP composites in the industry. For the autoclave process, the FRTP preform is laid on a tool, by hand or using AFP, and sealed in a vacuum bag assembly. The vacuum bag assembly can include release films, a bleeder, a breather, a vacuum bag and sealants. The assembly is then put into an autoclave where heat and pressure are applied to the FRTP composite [4,10]. Advantages of the autoclave process include being able to fabricate large and complex components, as well as the consistency in the quality of components produced [10]. However, the process is difficult to automate. Consolidation also takes longer due to the convection heating involved [4]. For FRTP composites with high performance thermoplastic matrix, high-temperature vacuum bagging materials, which are difficult to handle, need to be used [10]. 

In compression molding, the FRTP component is formed from a preform using a compression press and a mold set. Similar to the autoclave process, the preform can be made by hand layup or using AFP. Prior to molding, the preform can be preheated in an oven. The preform is then compressed between the upper and lower molds (or ‘core and cavity’) using a compression press to give the component its shape. Heat is also applied during this stage [4,11]. Advantages of compression molding include its short processing cycles and being suitable for automation [4,11]. However, FRTP components fabricated using automated compression molding tend to have defects caused by wrinkles and waviness in the fiber reinforcement [11].

Computer aided manufacturing processes such as AFP and FW have several advantages over the autoclave process and compression molding. One of the advantages is the better precision in the fiber orientation that can be achieved. AFP and FW can also include ISC to enable the fabrication of FRTP composite components in a single step, thus potentially reducing production times. The AFP and FW processes with ISC are discussed in more detail in the next sections.

### 2.1. Automated Fiber Placement

**Figure 1:**
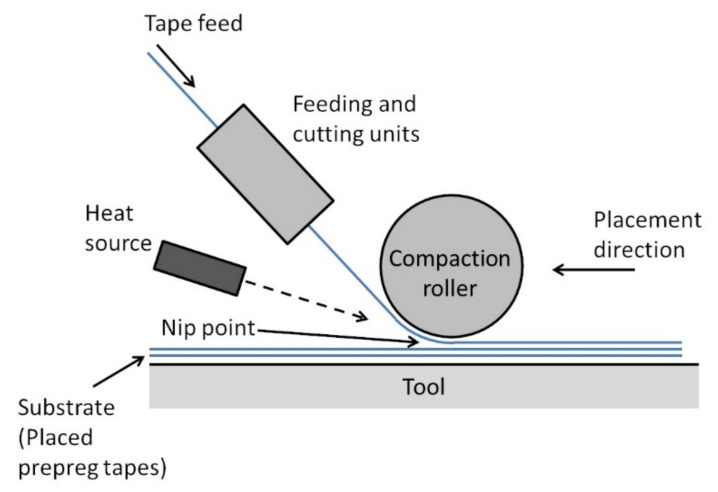
Automated fiber placement with in situ consolidation.

A schematic diagram of the AFP process for FRTP composites is shown in Figure 1. Strips of FRTP prepreg tapes are laid on a tool usually by a robot arm equipped with a fiber placement head. The feeding unit on the fiber placement head guides the prepreg tapes onto the tool. The tape is heated at the nip point by a heat source and pressed onto the substrate by a compaction roller for ISC. The tape is cut into strips of specified lengths by the cutting unit. The AFP process is controlled by a computer program to lay the FRTP prepreg tapes in the designed layup configuration. 

Advancements in AFP technology enabled the fabrication of FRTP composite components with improved fiber direction accuracy compared to conventional fabrication methods, such as hand layup, while reducing material wastage [12]. Advanced AFP machines allow for fiber placement speeds (or line speeds) of up to 3 m/s [13]. For the fabrication of large components, multiple tapes can be placed simultaneously to increase the productivity of the process [12]. For the fabrication of more complexed parts, the process is constrained by factors such as the size of the compaction roller, the arrangements of the feeding and cutting units, and the size of the heat source [13,14]. Some of the constraints can be overcome by designing the fiber placement head with features such as multiple compaction rollers with adjustable heights [13].

One of the major limiting factors to increasing the productivity of AFP processes is the incidence of manufacturing defects requiring inspections and corrections [13]. One strategy to alleviate the problem is to improve the defect detection using tools such as laser positioning systems [15] or online process monitoring methods such as thermographic imaging [13]. Sacco et al. presented a method using machine learning techniques in image processing to detect and classify defects in AFP processes [16]. Another strategy is to gain a better understanding of the effects of the defects on the performance of the FRP composite component and develop defect tolerant designs. Croft et al. conducted experiments to study the impacts of four different defect configurations, namely gap, overlap, half gap/overlap and twisted tow [17]. Nguyen et al. expanded on the study and investigated defects of various sizes [18]. Zhang et al. studied the tape wrinkling when placing prepreg tapes in curves and developed a set of placement criteria to avoid wrinkling [19]. Reviews on the effects of manufacturing defects in AFP and the methods for defect detection have been presented by Oromiehie et al. [20] and Sun et al. [21].

### 2.2. Filament Winding

**Figure 2:**
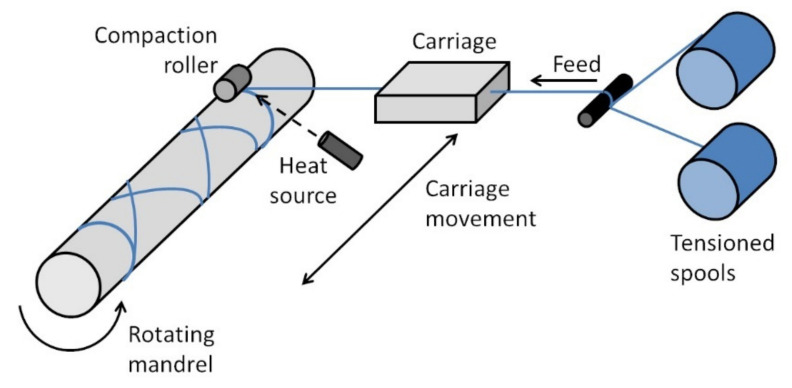
Filament winding with in situ consolidation.

Figure 2 shows a schematic of a FW setup with ISC. FRTP yarns or prepreg tapes are directed by the carriage and wound onto a rotating mandrel. For the fabrication of cylinders or pipes, a simple 2-axis FW machine (as shown in Figure 2) is sufficient. For the fabrication of parts with more complex geometry, such as pressure vessels with hemispherical end domes, a FW machine with more axes of motions, such as a robot arm with a FW head, is required. Similar to AFP, ISC is carried out using a heat source and a compaction roller. FW is a continuous process where cutting the FRTP yarns or prepreg tapes is not required during winding. As such, the yarns or tapes are usually tensioned throughout the process by tensioners.

FW has been used for decades to make axisymmetric FRP components, such as pipes, pressure vessels, pipe fittings and drive shafts [22,23]. Recently, advancements in robotics have made it possible to make components with more complexed shapes. Sorrentino et al. used FW to fabricate a helicopter “fork” (a structural component connecting the helicopter blade to the rotor) which is not axisymmetric [24]. The authors highlighted the considerations in designing the FW process, including the winding trajectory, the winding tool, the mold and the winding pattern. Beck et al. used FW to fabricate a 3D fiber skeleton which is then overmolded through injection molding to form the final part [25]. The continuous fibers in the 3D skeleton are oriented strategically to be aligned to the load path.

## 3. Materials

The fiber reinforcement in FRP composites is the main load bearing component. Fiber reinforcements that are commonly used include carbon, glass and aramid. Carbon fibers have very high stiffness and tensile strength, making them suitable for applications in the aerospace industry. The reinforcement fibers need to be surface treated with the proper sizing to ensure good fiber-matrix bonding in the FRP composite [26,27].

There are two types of thermoplastic polymers, namely semi-crystalline and amorphous thermoplastics. Semi-crystalline thermoplastics experience thermal transitions from a solid or glassy state to a rubbery state at the glass transition temperature, $T_{g}$, and subsequently to a liquid state at the melting temperature, $T_{m}$. On the other hand, amorphous thermoplastics only exhibits a $T_{g}$. Thermoplastic polymers are generally categorized into two groups based on their performances, namely low cost thermoplastics and high performance thermoplastics. Low cost thermoplastics include polypropylene (PP), polyethylene (PE) and polyamides, such as PA6, PA66, and PA12. High performance thermoplastics include polyether ether ketone (PEEK), polyether ketone ketone (PEKK), polyphenylene sulphide (PPS), polyetherimide (PEI) and high temperature polyimides (TPI) [4,8,28]. The mechanical properties of high performance thermoplastics are comparable or better than conventionally used thermosets such as epoxy. High performance thermoplastics also have high $T_{g}$ and high chemical resistance, allowing them to maintain their mechanical properties in harsh environments [8,9]. Gabrion et al. reported that a carbon/TPI composite retained its strengths in tensile and fatigue tests at temperatures up to 200 °C [28].

**Figure 3:**
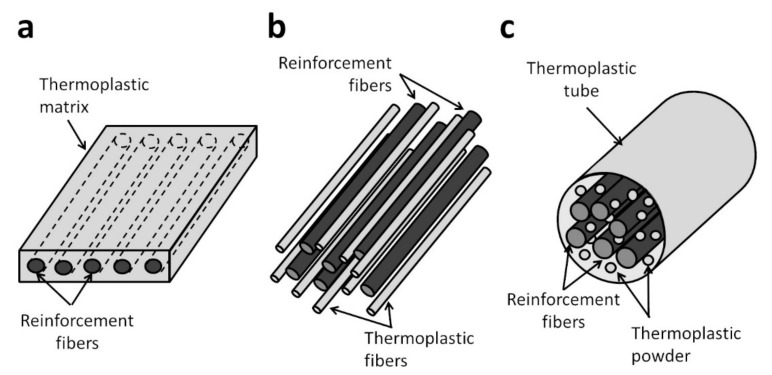
(**a**) Prepreg tape, (**b**) commingled yarn, (**c**) powder impregnated fibers.

For the processing of FRTP composites using FW and AFP, semi-finished forms of the material system are generally used. For AFP, prepreg tapes are commonly used. For FW, in addition to prepreg tapes, commingled yarns and powder impregnated fibers can also be used [29]. The various semi-finished forms of FRTP composites are shown in Figure 3.

Prepreg tapes are commonly produced through hot melt coating or pultrusion. High pressure is applied during the coating to ensure good thermoplastic impregnation. The resulting tapes are fully consolidated with very low void content [8]. Prepreg tapes with uniform tape width and smooth tape surfaces are suitable for use in FW and AFP. For FW and AFP using prepreg tapes, ISC only involves the bonding between the tapes and not impregnation, thus allowing for higher line speeds [29]. The FRTP component fabricated from prepreg tapes is also generally of better quality compared to using other semi-finished forms [4,29]. One disadvantage of prepreg tapes is their low flexibility making it difficult to form complex parts with them [4,8]. Prepreg tapes are also more expensive than commingled yarns and powder impregnated fibers [29,30]. Many FRTP materials, such as carbon/PEEK, carbon/PA6 and carbon/PPS, are currently available in prepreg tape form, and research on prepreg tapes for new material systems are ongoing. Iannone et al. proposed a hybrid prepreg tape consisting of carbon/PEEK tapes with PEI films added on the tape surfaces [31]. The hybrid prepreg tapes enable the fabrication of laminates with PEEK matrix at the desired degree of crystallinity and amorphous PEI at the interlaminar regions. 

Commingled yarns are produced by intermingling reinforcement fibers with thermoplastic fibers. The reinforcement and thermoplastic fibers need to be distributed evenly in the yarn to minimize the thermoplastic flow distance required to achieve good impregnation during consolidation. The intermingling process is usually performed using air jets at room or elevated temperatures [32]. More recently, the production of commingled yarns using an online hybrid melt spinning technique is developed [26]. The technique enables excellent intermingling of the fibers without damaging them, and sizing can be applied in the same process. The use of commingled yarns allows for the fabrication of FRTP components with uniform fiber volume content. Commingled yarns are also flexible and can be woven into highly drapeable fabrics which can be used in stamp forming [32,33,34]. For FW using commingled yarns, researchers have studied the use of various heat sources such as ultrasonic welding and hot gas torch for ISC [29,35,36].

Powder impregnated fibers are made of reinforcement fibers with fine dispersed thermoplastic powder. In some products, an outer tube made of the same thermoplastic material is added. However, the outer tube can lead to composite components with large matrix rich region [9]. Powder impregnated fibers are produced by passing the reinforced fibers through a fluidized bed (air fluidization) or an aqueous bath where thermoplastic powders are dispersed onto the fibers [8,37,38]. The deposition of the powders is aided by ionizing the powders or adding surfactants. This process enables thermoplastics with very high melt viscosities to be used in FRTP composites [9]. Similar to commingled fibers, powder impregnated fibers are flexible and can be woven into fabric forms [8,9]. 

Henninger et al. proposed an FW process with online impregnation [39,40]. The reinforcement fibers and thermoplastic matrix are used in their raw forms. The online impregnation is performed by passing the reinforcement fibers through porous impregnation wheels. Molten thermoplastic polymer is squeezed from the inside of the wheels through to the surface to impregnate the fibers. A hot air gun and a consolidation roller are used for ISC. Henninger et al. reported that the FW process with online impregnation led to lower costs overall due to the raw materials used [39]. By controlling the online impregnation parameters, the process can be used to fabricate FRTP components with varying fiber volume contents. However, the productivity is lower than FW processes using semi-finished FRTP products because the line speed is limited by the fiber pretension [40].

## 4. Heat Sources for In Situ Consolidation

Temperature and dwell time (or exposure time) are two related parameters crucial to the consolidation of FRTP composites. Low temperatures or short dwell times lead to insufficient bonding, whereas temperatures which are too high or dwell times that are overly long result in degradation and decomposition of the thermoplastic matrix [36,41,42]. The temperatures and dwell times achievable in the ISC process are limited by the heating rate and the heating zone of the heat source. Therefore, the choice of the ISC heat source affects the productivity of the AFP and FW processes, as well as the quality of the FRTP composite component fabricated. Many different types of heat sources have been used for ISC, including hot gas torch, infrared (IR) heater, laser and ultrasonic welder.

Hot gas torch has been widely used in AFP and FW due to its low capital cost [43,44]. It can also be attached to the AFP or FW heads easily [45]. Heat is transferred to the thermoplastic through forced convection. Factors affecting the heat transfer include the hot gas temperature, the gas flow rate and the distance of the nozzle to the nip point [45]. For consolidation requiring high temperatures, an inert gas, such as nitrogen, needs to be used to prevent oxidation. This results in a significant increase in the operating costs [43,44]. Another drawback of the hot gas torch is its low energy efficiency [43,46]. In order to improve the heating process, hot gas heating can be used together with methods such as preheating of the composite fibers or tapes [36,47] and using a heated mandrel or tool [48].

An IR heater is another inexpensive option for heating FRTP composites. They are also easy to operate. They have been used both for the preheating of incoming fibers or tapes and as the main heat source for ISC. For the latter, IR spot heaters can output more focused heating to achieve results that are better or comparable to a hot gas torch [44,49]. IR heaters transfer heat mainly through radiation. For the heating of powder impregnated fibers with outer tubes using IR heaters, only the outer surface is exposed to IR radiation. The heat needs to be transferred from the outer tube to the powders and the fibers through conduction or convection, thus leading to a delay in the heating process [47]. Another disadvantage of IR heaters is the inconsistent heating due to the residual heat in the heaters after they are switched off [50]. The heat transfer from IR heaters is also less focused, leading to inefficiencies [44]. 

Laser has been studied by many researchers for ISC. Lasers are characterized by their very high radiation intensity which allows fast heating at a localized point [43,44]. Researchers have used CO_2_ lasers for the consolidation FRTP composites such as carbon/PEEK and glass/PPS in early studies [42,43]. However, using CO_2_ lasers can cause burning and oxidizing at the surface of FRTP prepreg tapes as the radiation from CO_2_ lasers is absorbed by the thermoplastic matrix [44]. More recently, high powered lasers such as near infrared (NIR) diode lasers are used [51,52,53]. Diode lasers do not cause oxidation at the prepreg tape surface as the radiation from diode lasers is absorbed by the carbon fibers instead of the thermoplastic matrix [44]. The high power ratings of modern lasers (e.g., 3 kW [41]) compared to those used in early studies (e.g., 80 W [42]) allow AFP or FW to be performed at very high line speeds. Lasers are considerably more expensive than hot gas torch and IR heaters [43], thus they are generally reserved for the processing of FRTP composites requiring high temperatures, where other heating methods are considered insufficient. The implementation of a laser heating system is also more difficult. Additional processing parameters such as the laser angle, the laser beam profile and the distance between the laser and the nip point need to be taken into consideration [41,44,54]. Additionally, safety measures, such as having an enclosed environment, need to be implemented.

Ultrasonic welding is a relatively new technique for ISC of FRTP composites. The technique has been proven to be effective in the joining and repairing of thermoplastic-based components [55]. In ultrasonic welding, high frequency, low-amplitude ultrasonic vibration is applied to the thermoplastic surface through a sonotrode or horn to cause surface and intermolecular friction, resulting in the heating and melting of the thermoplastic polymer [35,56]. The advantages of ultrasonic welding include short processing time, good localized heating and low energy requirement [55,56]. For ultrasonic ISC, the horn also acts as a compaction unit to apply pressure onto the FRTP composite. Additional rollers can be added to increase the contact time [35]. Researchers have studied the ultrasonic ISC of FRTP composites in the forms of commingled yarns and prepreg tapes, with thermoplastic matrices including poly(ethylene terephthalate) (PET), PP and high density polyethylene (HDPE) [35,46,56,57]. One of the limitations of ultrasonic welding is the dependence of the heat transfer process on the FRTP material properties, including stiffness, hardness and damping response [55]. Rizzolo and Walczyk reported that ultrasonic welding is effective for the consolidation of carbon/PET but less so for glass/HDPE [46].

Heraeus Noblelight developed a new flashlamp heating system named, humm3^®^, based on pulsed light technology [58]. The humm3^®^ has very high heating rate and excellent temperature control. The heating performance of the humm3^®^ is comparable to high powered lasers. However, unlike lasers, there is no restrictive safety requirements for its application. Another advantage of the heating system is the small flashlamp head which allows it to be used for the fabrication of complex parts using AFP. Nguyen et al. used the flashlamp technology in the AFP of a hybrid material made for lightning protection, consisting of a FRTP prepreg layer, a copper layer (expanded or perforated foil) and a thermoplastic layer [59]. The line speed of 2400 mm/min was achieved for the process temperature of 380 °C. 

## 5. Modeling Heat Transfer

The heat transfer process is a crucial part of the ISC of FRTP composites. Heat transfer mechanisms are categorized into conduction, convection and radiation. For AFP and FW with ISC, due to the various mechanisms involved and the complexity of the process, heat transfer is often studied using numerical methods, such as finite element (FE) simulations and finite difference methods (FDMs). In numerical simulations, the temperature field for the material being studied is obtained by solving the heat equation, with the heat transfer between the material and the environment modeled as boundary conditions. From the energy balance principle, the general heat equation can be written as shown in Equation (1) [60]:

(1) $$\rho C\frac{\partial T}{\partial t}=-\nabla \cdot q+\phi$$

where ρ is the material density, C is the specific heat capacity of the material, T is temperature, t is time, q is the heat flux vector and ϕ is the volumetric rate of heat generation. For semi-crystalline thermoplastic polymers, the latent heat of fusion and heat related to crystallization can be modeled as a heat sink and a heat source respectively [61]. However, they are small compared to other heat exchange mechanisms involved and thus are often ignored [62,63]. The heat flux vector through the material, $q_{mat}$, can be obtained using Fourier’s law of heat conduction shown in Equation (2) [60]:

(2) $$q_{mat}=-k\nabla T$$

where k is the thermal conductivity of the material and ∇T is the temperature gradient. For FRTP composites, the thermal conductivity in the fiber direction is different from the thermal conductivity in the transverse direction. Therefore, the heat flux in the fiber and transverse directions need to be considered separately [62,64].

For the heat transfer boundary conditions, the heat flux vector, $q_{bc}$, is often described using a linear relation shown in Equation (3) [60]:

(3) $$q_{bc}\cdot n=h\left(T_{1}-T_{2}\right)$$

where n is the unit normal vector, h is the film heat transfer coefficient at the boundary, $T_{1}$ is the temperature of the material and $T_{2}$ is the temperature of the heat source or the environment. For heat transfer through conduction, radiation and convection, h is substituted by thermal conductivity at the boundary, $k_{bc}$, the radiation heat transfer coefficient, $h_{r}$, and the convection heat transfer coefficient, $h_{conv}$, respectively. For the radiation heat exchange between the material and the environment, $h_{r}$ is approximated by representing the material as a small body in a large enclosure. Using this representation, $h_{r}$ is defined as shown in Equation (4) [65]:

(4) $$h_{r}=4\epsilon \sigma T_{ref}^{3}$$

where ϵ is the emissivity of the material, σ is the Stefan-Boltzmann constant and $T_{ref}=\frac{1}{2}\left(T_{1}+T_{2}\right)$. For convection, two types of convection are considered, namely forced and free convection. Forced convection is the heat transfer between a surface and a flow of fluid that is driven by an external agency, such as a pump, whereas free convection is the heat transfer caused by fluid flow due to buoyancy [60,65]. For free convection in air, $h_{conv}$ is typically in the range of 1 to 50 W/(m^2^·K) [63,65].

For the modeling of ISC using forced convection heaters such as hot gas torch, many researchers used constant heat coefficient values ($h_{heater}$) to model the heat transfer between the heater and the FRTP material [66,67,68]. Tafreshi et al. discussed the determination of the $h_{heater}$ value for ISC using hot gas torch using two approaches, namely (i) calculating $h_{heater}$ from the Nusselt number and the Reynold number based on impinging jet theories, and (ii) obtaining $h_{heater}$ by considering the energy absorbed by the material and then fitting numerical calculations to experimental data [45]. The authors compiled the $h_{heater}$ values for different nozzle temperatures, hot gas flow rates, and nozzle-substrate distances for AFP with and without a roller. The data is useful for determining $h_{heater}$ for future studies on the topic. In a recent study, Zacherl et al. noted that assuming a constant $h_{heater}$ for the heating process using hot gas torch can lead to satisfactory predictions for the maximum temperature, but the predicted heating and cooling rates differ from experiments [69]. A similar observation was reported by Tafreshi et al. [68]. In particular, the cooling rate is important in determining the degree of crystallization of the thermoplastic in the ISC process. Therefore, Zacherl et al. introduced two distribution functions to model the $h_{heater}$ distributions along the length and width of the FRTP tape near the nip point [69]. Using the $h_{heater}$ distributions, the authors showed that the heating and cooling rates predicted agreed well with experiment. However, four additional parameters need to be determined through experimental data fitting, making the use of the distribution functions more complex.

For the modeling of ISC using radiant heating (lasers or IR heaters), the heat transfer from the heater to the FRTP composite is modeled using a heat flux distribution near the nip point. In earlier studies, researchers assumed a uniform heat flux distribution for ISC using radiant heating [61,70]. However, this is not an accurate representation due to factors such as reflection. The portion of radiation reflected can be calculated from the index of refraction of the material and the incident angle of the radiation using Fresnel’s relations [64,71]. For laser ISC, Stokes-Griffin and Compston presented a model which includes a spatial emittance function to simulate the divergent beam and a micro-half-cylinder surface model to represent the surface of the FRTP tape for determining reflections [54]. The authors reported that the heat flux distribution predicted by the model agreed well with experiments. Baho et al. used a ray-tracing algorithm to determine the incident beam distribution for laser ISC and study the effect of reflected beams on the heat flux distribution near the nip point [64]. The authors reported that the laser heat flux is underestimated by 38% if reflection is neglected, 19% if only the first reflection is considered, 6% if reflections up to the second reflection are considered and 2% if reflections up to the third reflection are considered.

Several approaches have been used by researchers to model the relative movement of the heat source to the FRTP material. Many researchers modeled the ISC process using the nip point as the Eulerian reference and including a velocity vector in the heat equation to simulate the mass flow in the material [61,62,63,64,72]. This approach is limited to modeling ISC at a constant line speed. Fricke and Fischer modeled the moving heat source in ISC using a boundary condition that is a function of time and space [73]. A script was used to generate a table to activate or deactivate the boundary condition at different locations and time steps in the process simulation. Another approach used by Liu et al. in their study involved the use of elements with life and death settings in FE simulation [66]. The tape laying process is simulated by having elements representing the tape in the death state initially and activating the elements at the appropriate time steps.

## 6. Modeling FRTP Consolidation

### 6.1. Consolidation of Prepreg Tapes

The determining factor in the consolidation of FRTP composites made using prepreg tapes is the bonding between the tapes, as full consolidation is already realized within the tapes. Two conditions need to be met: (i) intimate contact between the FRTP tapes or layers and (ii) autohesion or polymer chain inter-diffusion [44,74,75]. The models developed to study the two conditions are discussed next.

#### 6.1.1. Intimate Contact Model

**Figure 4:**
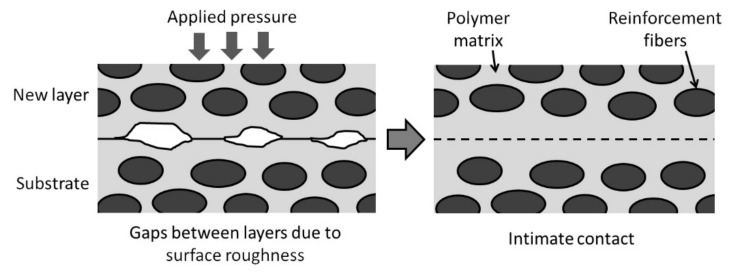
Establishing intimate contact at the interface between FRTP layers.

Figure 4 shows the schematic diagram of the interface between a newly placed FRTP layer and the substrate. Initially, gaps exist between the layers due to the rough surfaces of both the new layer and the substrate. The gaps prevent the autohesion of the thermoplastic at the interface [74,75]. Pressure needs to be applied to close the gaps and bring the surfaces together. The thermoplastic matrix also needs to be heated to lower its viscosity.

**Figure 5:**
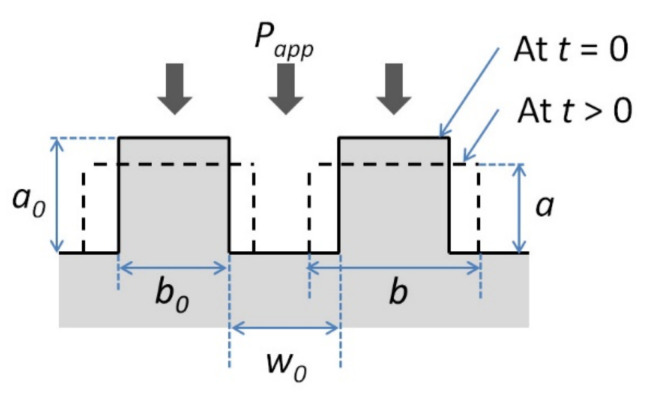
Simplified surface representation used by Lee and Springer for their intimate contact model.

Dara and Loos developed an intimate contact model to study the effects of pressure, temperature and contact time on the degree of intimate contact, $D_{ic}$ [74]. $D_{ic}$ is defined as the ratio of the area in contact to the total area of the interface. This follows that $D_{ic}=1$ when intimate contact is achieved. In their model, Dara and Loos used a statistical distribution to describe the roughness at the interface. The viscoelastic behavior of the thermoplastic is modeled as a squeezing flow of homogenous fluid between two plates. Lee and Springer simplified the model by representing the surface roughness as a series of rectangles with uniform width ( $b_{0}$) and height ($a_{0}$) spaced evenly apart ($w_{0}$) [76]. The simplified representation of the surface is given in Figure 5 [76].

Using this simplification, $D_{ic}$ is given by Equation (5) [76]:

(5) $$D_{ic}=\frac{1}{1+\frac{w_{0}}{b_{0}}}{\left[1+\frac{5P_{app}}{\mu_{mf}}\left(1+\frac{w_{0}}{b_{0}}\right){\left(\frac{a_{0}}{b_{0}}\right)}^{2}t\right]}^{1/5}$$

where $P_{app}$ is the applied pressure, $\mu_{mf}$ is the viscosity of the matrix-fiber mixture, and t is time. Here, it is assumed that $P_{app}$ and $\mu_{mf}$ are constant over time. Mantell and Springer later expanded the model to include changes of $P_{app}$ and $\mu_{mf}$ with time [77]. The resulting more generalized expression for $D_{ic}$ is given by Equation (6) [77]:

(6) $$D_{ic}=\frac{1}{1+\frac{w_{0}}{b_{0}}}{\left[1+5\left(1+\frac{w_{0}}{b_{0}}\right){\left(\frac{a_{0}}{b_{0}}\right)}^{2}\int_{0}^{t_{c}}\frac{P_{app}}{\mu_{mf}}dt\right]}^{1/5}$$

where $t_{c}$ is the contact time in which pressure is applied.

Yang and Pitchumani proposed an intimate contact model using fractal Cantor set construction to represent the surface roughness of FRTP prepreg tapes [78]. The Cantor set surface consists of generations of asperities with decreasing sizes controlled by a scaling factor f. The intimate contact process is modeled as the flattening of the asperities generation by generation following the squeeze flow model. In other words, the flattening of the (n+1)th generation asperities is completed before the (n)th generation asperities start to deform and become flattened. The degree of intimate contact for the (n)th generation asperities at time t, $D_{ic}^{n}\left(t\right)$, is given by Equation (7) [78]:

(7) $$D_{\mathrm{ic}}^{n}\left(t\right)=\frac{1}{f^{n}}\;{\left[\frac{5}{4}{\left(\frac{h_{0}}{L_{0}}\right)}^{2}\frac{f^{\left(\frac{2nD}{2-D}+n+4\right)}}{{\left(f+1\right)}^{2}}\int_{t_{n+1}}^{t}\frac{P_{app}}{\mu }dt+1\right]}^{1/5},\;t_{n+1}\leq t\leq t_{n}$$

where μ is the viscosity of the thermoplastic matrix, $L_{0}$ is the total length of the Cantor set surface, $h_{0}$ is the height of the first-generation asperities and D is the fractal dimension of the Cantor set surface. For the models developed in [76,77], the parameters describing the surface roughness are obtained through experimental data fitting. In contrast, the parameters $L_{0}$, $h_{0}$, D and *f* required for Yang and Pitchumani’s model can be obtained from surface roughness measurements [78].

The models shown in Equations (5)–(7) have been used by researchers to study the FW and AFP processes with ISC [41,72,79]. Yassin and Hojatti highlighted the concerns that need to be accounted for when applying the intimate contact models [44]. For FW and AFP with ISC, the pressure is applied through the compaction roller. Factors such as the size and material (rigid or deformable) of the roller and the line speed affect the consolidation pressure applied and the contact time. Besides this, the temperature of the FRTP composite, and in turn the composite viscosity, are also affected by the roller when they are in contact. These factors should be taken into consideration when using the models to study the ISC process, especially for processes with short contact times.

#### 6.1.2. Autohesion Model

**Figure 6:**
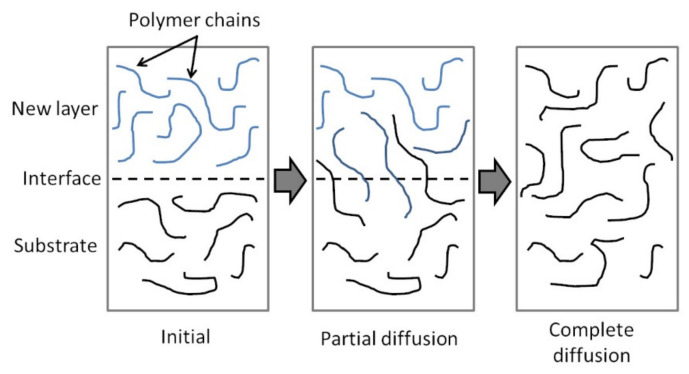
Polymer chain diffusion in the autohesion process.

Figure 6 shows a schematic diagram of the autohesion or healing process for the consolidation of thermoplastic polymers. When a new thermoplastic layer is brought into intimate contact with the substrate and heat is applied, polymer chain inter-diffusion across the interface can occur. Over time, enough polymer chain inter-diffusion take place and the interface becomes indistinguishable from the bulk polymer [74,75]. This results in strong bonding between the thermoplastic layers. Autohesion can occur at temperatures above $T_{g}$ for amorphous thermoplastics and above $T_{m}$ for semi-crystalline thermoplastics [80,81]. 

The diffusion of polymer chains is modeled based on the reptation theory detailed by De Gennes [82]. A polymer chain entangled with many other chains is restricted to wriggling motions in a tube-like region at small time scales. At larger time scales, the ends of the polymer chain can move away from the tube, forming minor chains. Eventually, the polymer chain has a new position out of the initial tube. The time to reach this state is the renewal or reptation time, $t_{r}$ [82,83]. The degree of autohesion, $D_{ah}$, is defined as the ratio of the interfacial bond strength to the fracture strength of the virgin thermoplastic polymer. Kim and Wool considered the interfacial bond strength as a function of the length of minor chains through the interface [83]. For time $t<t_{r}$, $D_{ah}$ was found to be proportional to $t^{1/4}$ and $M^{4/3}$, where M is the molecular weight of the polymer.

Yang and Pitchumani proposed an autohesion model for non-isothermal condition [80]. Bonding at the interface is dependent on the critical entanglement molecular weight, $M_{C}$. $M_{C}$ is a function of the number of monomers, the polymer chain length, the monomer weight, the number of C-C bonds per monomer, the bond length and the characteristic ratio [84]. For thermoplastic polymers with molecular weight $M>8M_{C}$, the maximum bond strength is reached at the welding time, $t_{w}$ with $t_{w}<t_{r}$. This is the case for most engineering thermoplastic polymers. The degree of autohesion for non-isothermal healing is given by Equation (8) [80]:

(8) $$D_{ah}\left(t\right)={\left[\int_{0}^{t}\frac{1}{t_{w}\left(T\right)}dt\right]}^{\frac{1}{4}}$$

where T is the temperature. For polymers with $M<8M_{C}$, the welding time is the same as the reptation time ( $t_{w}=t_{r}$). $t_{w}$ is dependent on T, with their relationship described by an Arhennius law [80]. At higher temperatures, the polymer chains can diffuse more easily, leading to shorter welding times.

#### 6.1.3. Bonding Model

For the bonding of FRTP prepreg tapes, intimate contact has to occur before autohesion can take place. Yang and Pitchumani described the bonding process as the healing of incremental intimate contact areas. The degree of bonding, $D_{b}$, is thus given as shown in Equation (9) [85]:

(9) $$D_{b}\left(t_{b}\right)=D_{ic}\left(0\right)\cdot D_{ah}\left(t_{b}\right)+\int_{0}^{t_{b}}D_{ah}\left(t_{b}-t\right)\cdot \frac{dD_{ic}\left(t\right)}{dt}dt$$

where $t_{b}$ is the total bond time, $D_{ic}\left(0\right)$ is the initial intimate contact area and ( $t_{b}-t$*)* is the time available for autohesion for an incremental intimate contact area. For cases where the time required to achieve full intimate contact is much larger than the time required for autohesion (e.g., as reported in [41]), $D_{b}$ is taken to be the same as $D_{ic}$.

### 6.2. Consolidation of Commingled Yarns

The consolidation process of FRTP composites made from commingled yarns involves the impregnation of fiber bundles by the thermoplastic matrix, in addition to the intimate contact and autohesion of the thermoplastic. The impregnation step is usually the limiting step [86], thus researchers have focused on the impregnation step in studying the consolidation of commingled yarns.

**Figure 7:**
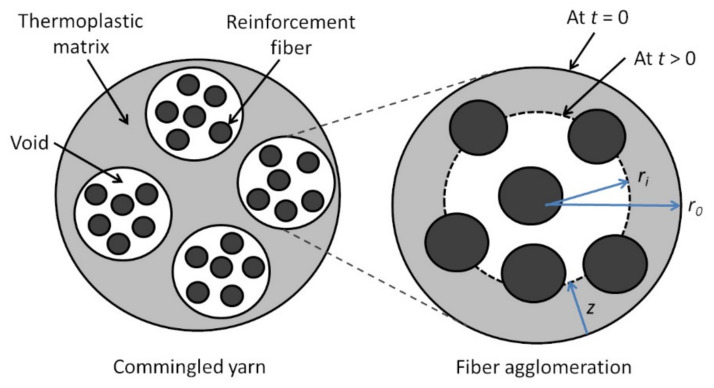
Cross section of commingled yarn modeled as a group of fiber agglomerations.

Klinkmuller et al. developed an impregnation model for commingled yarns [87]. The commingled yarns are modeled as consisting of several fiber agglomerations of the same size surrounded by the thermoplastic matrix (Figure 7). Consolidation is achieved when the voids in the fiber agglomerations are filled by the matrix. 

The Darcy’s law is used to describe the flow of the thermoplastic polymer through the reinforcement fibers (functioning as a porous bed). For the transverse permeability through the reinforcement fibers, the model presented by Gutowski et al. is used for cases with high fiber volume content [88]. The impregnation time, t, required to reach a penetration length, z, is given by Equation (10) [87]:

(10) $$t=\frac{2\mu k_{zz}z^{2}\left(\frac{V_{a}}{V_{f}}+1\right)}{r_{f}^{2}\left(p_{a}-p_{0}\right){\left(\sqrt{\frac{V_{a}}{V_{f}}-1}\right)}^{3}}$$

where:

- μ is the viscosity of the thermoplastic matrix dependent on the temperature through an Arhennius law,
- $V_{a}$ is maximum fiber volume content (0.83 for carbon fibers [88]),
- $V_{f}$ is the pressure dependent fiber volume content,
- $r_{f}$ is the radius of the reinforcement fiber,
- $p_{a}$ is the applied pressure,
- $p_{0}$ is the atmospheric pressure, and
- $k_{zz}$ is the transverse permeability constant through the fibers.

For fiber agglomerations with circular cross sections (Figure 7), z is related to void content, $X_{v}$, by Equation (11) [87]:

(11) $$z=r_{0}-\sqrt{\frac{X_{v}A_{t}}{\pi n\left(1-V_{f}\right)}}$$

where $r_{0}$ is the radius of the fiber agglomeration, n is the number of fiber agglomerations in the yarn and $A_{t}$ is the total cross sectional area of the commingled yarn.

For fiber agglomerations with rectangular cross sections, the relationship between z and $X_{v}$ is given by Ye et al. [89], and also reported by Friedrich [32]. The relationship is shown in Equation (12) [32,89]:

(12) $$z=\frac{h_{0}\left(1-V_{f}\right)-h_{1\left(z,0\right)}\;X_{v}}{\left(1-V_{f}\right)\left(1-X_{v}\right)}$$

where $h_{0}$ is the half height of the fiber agglomeration and $h_{1\left(z,0\right)}$ is the half height of the total agglomeration, which includes the surrounding thermoplastic matrix, at time, t=0. The models (Equations (10)–(12)) can give satisfactory predictions for the impregnation time required to achieve a target void content, but require many parameters determined through experiments and empirical data fitting. In particular, the experiments required to determine $k_{zz}$ is difficult to carry out [88].

Another model for the consolidation of commingled fibers is proposed by Bernet et al. [33,86]. The commingled yarn is also modeled as a group of fiber agglomerations, but the agglomerations can have different sizes. The fiber volume fraction, $V_{f}$, is assumed to be constant after some pressure is applied, thus permeability of the fiber bed, $K_{P}$, is also constant. The position of the thermoplastic flow front, $r_{i}$, is used in the model (Figure 7). For unimpregnated fiber agglomerations, the voids are considered open pores. As impregnation progresses, the thermoplastic matrix connects the fibers causing air to be trapped in the voids. At this stage, the voids are considered closed pores. A parameter, $r_{c}$, is defined such that when $r_{i}\geq r_{c}$, voids are open pores, and when $r_{i}<r_{c}$, voids are closed pores. Using the Darcy’s law, the relationship between $r_{i}$ and time, t, is given by Equation (13) [33,86]:

(13) $$\Delta t=\frac{\mu \left(1-V_{f}\right)}{K_{P}\left(P_{a}+P_{c}-P_{g}\left(r_{i}\right)\right)}\left[\frac{r_{i}^{2}}{2}\ln\left(\frac{r_{i}}{r_{0}}\right)-\frac{R_{i}^{2}}{2}\ln\left(\frac{R_{i}}{r_{0}}\right)-\frac{r_{i}^{2}}{4}+\frac{R_{i}^{2}}{4}\right]$$

where $P_{c}$ is the capillary pressure (defined as positive when enhancing flow), $P_{a}$ is the applied pressure, $R_{i}$ is the thermoplastic flow front before the time increment, Δt, and μ is the viscosity of the thermoplastic matrix. $P_{g}\left(r_{i}\right)$ is the internal void pressure given by Equation (14) [33,86]:

(14) $$P_{g}\left(r_{i}\right)=P_{0}\;\mathrm{for}\;r_{i}\geq r_{c}\;P_{g}\left(r_{i}\right)=P_{0}{\left(\frac{r_{c}}{r_{i}}\right)}^{2}\;\mathrm{for}\;r_{i}<r_{c}$$

where $P_{0}$ is the ambient pressure in the fiber agglomeration before void closing. Equation (13) is obtained through numerical integration where $P_{g}\left(r_{i}\right)$ is assumed to be constant over the time increment, Δt. Total impregnation time for ri<rc is the sum of the time increments. $P_{c}$ is small compared to $P_{a}$ and can usually be neglected [86].

The fiber agglomerations are grouped according to the agglomeration size. For agglomeration size group j, $N_{a}^{j}$ is the number of agglomerations in the group and $r_{i}^{j}$ is the thermoplastic flow front. The void content of the yarn, $X_{v}$, is then computed using Equation (15) [33,86]:

(15) $$X_{v}=\frac{\sum_{j=1}^{n}\pi N_{a}^{j}r_{i}^{j2}\left(1-V_{f}\right)}{A_{t}+\sum_{j=1}^{n}\pi N_{a}^{j}r_{i}^{j2}\left(1-V_{f}\right)}$$

where n is the number of agglomeration sizes and $A_{t}$ is the cross section area of a fully consolidated yarn. The number of agglomerations and their sizes can be determined from microscopic observations of the commingled yarn. For the determination of $K_{P}$, Bernet et al. used Gebart’s model [90] given in Equation (16):

(16) $$K_{P}=C_{1}{\left(\sqrt{\frac{V_{f,max}}{V_{f}}-1}\right)}^{2.5}R_{f}^{2}$$

where $C_{1}$ is a constant, $V_{f,max}$ is the maximum fiber volume fraction corresponding to zero permeability and $R_{f}$ is the fiber radius. $C_{1}$ and $V_{f,\;max}$ are related to the fiber arrangement. Bernet et al. used their model to study the consolidation of carbon/PA12 commingled fibers [33,86]. The authors showed that the model is applicable for processes with consolidation times in the order of 1 min, 0.1 min and 10 s [33].

Thomann and Ermanni expanded on the model proposed by Bernet et al. [34]. The authors used a logarithmic normal distribution to better describe the fiber distribution within a commingled yarn with good blending. The frequency of occurrence for a fiber agglomeration of size j is given by Equation (17) [34]:

(17) $$\phi \left(N_{a}^{j}\right)=\frac{1}{s\sqrt{2\pi }\sum_{j=1}^{n}N_{a}^{j}}\exp\left(-\frac{1}{2}{\left(\frac{\ln\left(N_{f}^{j}\right)-\zeta^{*}}{s}\right)}^{2}\right)$$

where $N_{f\;}^{j}$ is the number of fibers in the agglomeration of size group j, $s^{2}$ is the variance and $\zeta^{*}$ is the predictand of $\ln\left(N_{f}^{j}\right)$. The parameters defining the fiber distribution are estimated from microscopic observations of the comminged yarn. Thomann and Ermanni used the model to study the stamp forming of FRTP composite components made from carbon/PA12 and carbon/poly(butylene-terephthalate) (PBT) commingled yarns [34].

### 6.3. Consolidation of Powder Impregnated Fibers

Similar to commingled fibers, the impregnation of the reinforcement fibers by the thermoplastic matrix is the limiting step in the consolidation of powder impregnated fibers.

Ye et al. developed a model to study the consolidation of powder impregnated fibers [91]. The thermoplastic flow is assumed to occur only in the radial direction (transverse flow through the fiber bed). Therefore, the impregnation process is modeled similar to that of commingled yarns (as reported in [32,87,89]) with a representative fiber agglomeration or tow. Using Darcy’s law, the time, t, required for the thermoplastic flow front to reach a position $r_{i}$ is given by Equation (18) [91]:

(18) $$t=\frac{\mu r_{0}^{2}}{4K_{P}\left(p_{a}-p_{0}\right)}\left[2{\left(\frac{r_{i}}{r_{0}}\right)}^{2}\ln\left(\frac{r_{i}}{r_{0}}\right)+1-{\left(\frac{r_{i}}{r_{0}}\right)}^{2}\right]$$

where $r_{0}$ is the radius of the fiber tow, $p_{0}$ is the pressure within the tow (taken as atmospheric pressure), $p_{a}$ is the applied pressure, $K_{P}$ is the permeability and μ is the viscosity of the thermoplastic matrix. The void content, $X_{v}$, can be calculated from $r_{i}$ using Equation (19) [91]:

(19) $$X_{v}=\frac{\pi r_{0}^{2}{\left(\frac{r_{i}}{r_{0}}\right)}^{2}\left(1-V_{f}-V_{mp}\right)}{\pi r_{0}^{2}\left(1+{\left(\frac{r_{i}}{r_{0}}\right)}^{2}\left(1-V_{f}-V_{mp}\right)\right)}$$

where $V_{f}$ is the fiber volume fraction and $V_{mp}$ is the volume fraction of the matrix powder. Ye et al. used the Carman-Kozeny equation to determine $K_{P}$ [91]. The authors reported good correlation between the model predictions and experimental results in their study on glass/PP composites [91]. 

Steggal-Murphy et al. used a similar model to that of Ye et al. [92]. In their study, Steggal-Murphy et al. investigated the consolidation of an FRTP composite made with glass fiber fabric as reinforcement and HDPE powders as matrix. A rectangular cross section was used to model a powder impregnated fiber bundle. The deformation of the fiber tow is assumed to be negligible. The authors used an empirical approach to determine the ratio of K/μ, where K is the permeability of the fiber bundle and μ is the viscosity of the thermoplastic. The consolidation was divided into two stages, namely the ramp stage where pressure is increased to a setpoint and the dwell stage where pressure is held constant. The authors reported that the model gave good predictions of the laminate void content for consolidation carried out at low applied pressure (0.345 MPa). However, for consolidation performed at high pressure (0.690 MPa), the void content is overestimated by the model. This is because the effects of fiber tow deformation become significant when the applied pressure is high [92].

**Figure 8:**
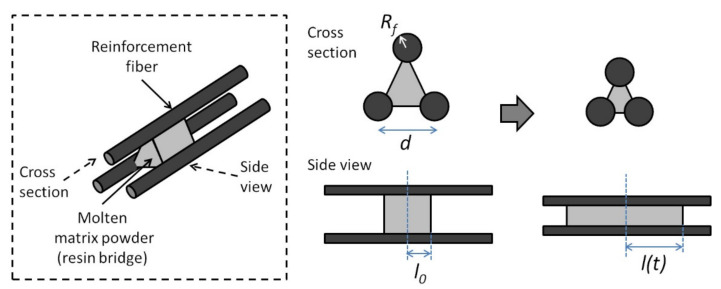
Model of resin bridge between fibers during the consolidation of powder impregnated fibers.

Connor et al. used a different approach in their impregnation model for powder impregnated fibers [93]. During consolidation, the thermoplastic matrix powders melt and form resin bridges between the reinforcement fibers. The impregnation process is modeled as the thermoplastic flow along the length of the fibers (axial flow), as shown in Figure 8. The distance between the reinforcement fibers, d, decreases as impregnation occurs. The assumptions made by the authors include: (i) transverse thermoplastic flow through the fibers is negligible compared to the axial flow, (ii) the center of the molten matrix powder is fixed and its volume is constant, and (iii) all of the resin bridges can be modeled using the same geometry.

For the consolidation process, the applied pressure ($P_{a}$) is counteracted by the capillary pressure ($P_{C}$), viscous pressure from the thermoplastic ($P_{v}$) and the spring-like pressure from the fiber bundle ($P_{s}$). $P_{s}$ is due to the compression of the fiber bundle as reported by Gutowski et al. [88]. Connor et al. applied the Hagen-Poiseuille equation to relate the thermoplastic flow to $P_{v}$. The flow rate is then expressed as shown in Equation (20) [93]:

(20) $$\frac{d}{dt}\left(\frac{l}{L}\right)=\frac{27}{128\mu }{\left(\frac{R_{f}}{R_{m}}\right)}^{6}{\left(\frac{V_{m}}{V_{f}}\right)}^{4}{\left(\frac{L}{l}\right)}^{4}\left(P_{a}-P_{s}-P_{c}\right)$$

where l is the time dependent thermoplastic half length (Figure 8), L is the half length of the molten thermoplastic powder when consolidation is completed (l=L at full consolidation), $R_{f}$ is the radius of the reinforcement fiber, $R_{m}$ is the thermoplastic powder radius, μ is the thermoplastic viscosity, $V_{f}$ and $V_{m}$ are the final fiber and matrix volume contents. $P_{c}$ is usually much smaller than $P_{a}$ and thus can be neglected. For the carbon/PEEK and carbon/PEI composites studied, the authors also found that $P_{s}$ was insignificant compared to $P_{a}$. In cases where $P_{c}$ and $P_{s}$ are small, the time to reach full consolidation, $t_{1}$, can be determined from Equation (21) [93]:

(21) $$\left(\frac{t_{1}P_{a}}{\mu }\right)=\frac{128}{135}{\left(\frac{V_{f}}{V_{m}}\right)}^{4}{\left(\frac{R_{m}}{R_{f}}\right)}^{6}$$

For cases where $P_{c}$ or $P_{s}$ is comparable to $P_{a}$, Equation (20) needs to be integrated numerically. In the model, the effect of different wetting behaviors in different material systems is absent as $P_{c}$ is neglected. However, the authors stressed that the wetting behaviors are important for the fiber-matrix bonding and need to be taken into consideration in studying the consolidation of powder impregnated fibers [93].

## 7. Modeling Crystallization and Other Aspects of FRTP In Situ Consolidation

For FRTP with semi-crystalline thermoplastic matrices, the morphology of the thermoplastic affects the mechanical properties of the composite. Factors affecting the morphology include the degree of crystallinity, spherulite size and crystalline orientation [8]. Many researchers use the degree of crystallinity as a means of studying the morphology [51,53,94,95]. For PEEK, tensile strength and stiffness increases with increasing crystallinity but fracture toughness decreases at the same time [8]. Therefore, the consolidation process, especially the cooling rate, needs to be controlled properly to achieve the desired crystallinity [8,69]. 

Ozawa derived an expression for non-isothermal crystallization given in Equation (22) [96]:

(22) $$\log\left[-\ln\left(1-c_{r}\right)\right]=\log X\left(T\right)+n\log\left(\frac{dT}{dt}\right)$$

where $c_{r}$ is the relative crystallinity, T is the temperature, t is time, $X\left(T\right)$ is the cooling function, and n is a constant. $X\left(T\right)$ and n can be obtained by analyzing differential scanning calorimetry (DSC) curves of the crystallization process occurring at different cooling rates. The crystallinity, $c_{a}$, is related to $c_{r}$ through Equation (23) [76]:

(23) $$\frac{c_{a}}{c_{r}}=\frac{H_{T}}{H_{U}}$$

where $H_{T}$ is the total heat of crystallization at a given cooling rate and $H_{U}$ is the theoretical ultimate heat of crystallization for the thermoplastic. Researchers have used the Ozawa model to simulate the crsytallization process of FRTP composites such as carbon/PEEK [76,97]. For the APC-2 carbon/PEEK composite, n=0.8, $X\left(T\right)=\exp\left(-0.037\;T+11.3\right)$, and the ratio $H_{T}/H_{U}$ can be calculated using $H_{T}/H_{U}=-0.03\ln\left(dT/dt\right)+0.42$ [76,97].

Another non-isothermal crystallization model was derived by Choe and Lee [98]. The model is based on phase transition kinetics formulated by Tobin, which includes the growth site impingement phenomena [99,100,101]. The non-isothermal crystallization expression derived by Choe and Lee is given in Equation (24) [98]:

(24) $$\begin{array}{c} \dot{\alpha }\left(t\right)=\kappa_{1}\exp\left(-\frac{3E_{d}}{RT}\right)\exp\left(-\frac{3\psi_{1}T_{m}^{0}}{T\left(T_{m}^{0}-T\right)}\right)t^{2}{\left[1-\alpha \left(t\right)\right]}^{2}\\ +\kappa_{2}\exp\left(-\frac{4E_{d}}{RT}\right)\exp\left(-\frac{\left(3\psi_{1}+\psi_{2}\right)T_{m}^{0}}{T\left(T_{m}^{0}-T\right)}\right)\\ \cdot {\left[1-\alpha \left(t\right)\right]}^{2}\int_{0}^{t}{\left(t-\omega \right)}^{2}\left[1-\alpha \left(\omega \right)\right]d\omega \end{array}$$

where *α*(*t*) is the relative crystallinity at time *t*, T is the temperature, $T_{m}^{0}$ is the equilibrium melting temperature, $E_{d}$ is the activation energy of diffusion of crystallization segments across the phase boundary, $\psi_{1}$ is a constant related to the free energy of formation of a critical nucleus, $\psi_{2}$ is a constant related to the free energy of formation of a growth embryo, $\kappa_{1}$ and $\kappa_{2}$ are kinematic parameters. The expression is applicable for crystallization through the formation 3D spherulites. The first part of Equation (24) describes the rate of crystallization through heterogenous nucleation and growth, while the second part gives the rate of crystallization through homogenous nucleation and growth. Choe and Lee varied the temperatures and cooling rates in DSC to control the crystallization mechanisms of PEEK. The values of $T_{m}^{0}$, $E_{d}$, $\kappa_{1}$, $\kappa_{2}$, $\psi_{1}$, $\psi_{2}$ for PEEK were then determined analyzing the DSC results with the use of regression methods [98]. 

In a study on AFP with laser ISC, Sonmez and Hahn used Choe and Lee’s model to simulate the crystallization in the FRTP tape in the cooling phase [61]. For the crystal melting process occurring in the heating phase, the model proposed by Maffezzoli et al. [102] was used. From the model, the crystallinity at time t, $c_{a}\left(t\right)$, is given by Equation (25) [61,102]:

(25) $$c_{a}\left(t\right)=c_{a}\left(t_{init}\right){\left(1-0.5\int_{t_{init}}^{t}\bar{K}dt\right)}^{2}$$

where $t_{init}$ is the initial time and $\bar{K}$ is given by $\bar{K}={\bar{K}}_{0}\exp\left(-E_{am}/RT\right)$. For APC-2 composite, the constant ${\bar{K}}_{0}=5.05\times 10^{31}\;s^{-1}$ and the activation energy for melting, $E_{am}$, is 397 kJ/mol [72,77]. Sonmez and Hahn’s approach was also used by Song to simulate the crystallization process in FW of carbon/PEEK with ISC using hot gas torch [72].

Gordnian used a ‘semi model-free’ approach in modeling the crystallization and melt kinetics of the carbon/PEEK composite [103]. Multiple isothermal and non-isothermal DSC experiments were carried out. Using the DSC results, Gordnian plotted the ‘iso-conversional’ graphs for the crystallization rate to study its dependence on crystallinity and the temperature. An empirical model was then introduced with the model parameters determined through experimental data fitting. For cases where the crystallinity is below 0.001, an induction time need to be calculated to determine the onset of the crystallization process. Gordnian also studied the melt kinetics using the same procedure. Two peaks were observed in the melting DSC experiments. The author deduced that recrystallization and melting occurred simultaneously in the process, resulting in the two peaks observation. A master melt curve was then obtained using the DSC results and the results calculated from the crystallization model. The combined model was shown to give good predictions for the crystallinity in isothermal and non-isothermal crystallizations and melting.

Besides crystallinity, researchers have also modeled other aspects of FRTP composites fabricated using AFP and FW with ISC. Schlottermuller et al. studied the thermal residual stresses in glass/PP specimens made using FW with hot gas torch ISC [104]. The thermal expansion coefficients are included in the stress-strain submodel to calculate the stress distributions across the composite layers. Dedieu et al. used the same approach to study the residual stresses in carbon/PEEK composites fabricated using FW with laser ISC [105]. Nam and Seferis presented a model for the degradation kinetics in thermoplastic composites [106]. The degradation model is defined by a rate constant, $k\left(T\right)$, and a conversion dependence function, $f\left(\alpha \right)$. The temperature dependence of $k\left(T\right)$ is described by an Arrhenius expression. For $f\left(\alpha \right)$, the effects of different reaction mechanisms are included using weighting factors. Nam and Seferis obtained the kinetic parameters for the degradation of PEEK from thermogravimetric analysis (TGA) experiments and showed that the model gave good predictions. The model developed by Nam and Seferis was adopted by Sonmez and Hahn in their studies on AFP with ISC [61,107].

## 8. Studies on Factors Affecting In Situ Consolidation Quality

Many researchers have carried out experimental and numerical studies on the fabrication of FRTP composites using AFP and FW with ISC. Many researchers compared the composite components fabricated using AFP and FW with components fabricated using more established methods for FRTP composites, such as compression molding and autoclave. The quality of the fabricated component is evaluated by its void content as well as mechanical properties measured from tests such as the short beam shear (SBS) test, peel tests, bending tests and delamination tests (such as the double cantilever beam (DCB) test). 

Comer et al. compared the mechanical performance of carbon/PEEK laminates fabricated using AFP with laser ISC with laminates fabricated using autoclave [51]. Carbon/PEEK prepreg tapes with a width of 12 mm were used in the AFP process. For the laminates fabricated using autoclave, wide carbon/PEEK tapes (width of 150 mm) were used to make the pre-forms by hand lay-up. The pre-forms were then sealed in a high temperature vacuum bag and consolidated in an autoclave. The specimens fabricated using the AFP process have higher interlaminar fracture toughness compared to the autoclave specimens. However, the AFP specimens have lower crystallinity (17.6%) than the recommended level (33%). This led to the specimens having lower flexural strength, interlaminar shear strength, and open-hole compression strength compared to autoclave specimens (crystallinity 40%). The authors suggested the high cooling rate and insufficient through thickness heating as two possible causes for the low crystallinity in the specimens fabricated using laser ISC.

Hoa et al. studied the fabrication of carbon/PEEK laminates using AFP with ISC using a hot gas torch [48]. Carbon/PEEK prepreg tapes were used in the study. The mechanical properties of carbon/PEEK laminates consolidated using the autoclave method from literature were used for comparison. The authors reported increased fiber waviness in the laminates fabricated using AFP compared to laminates fabricated using autoclave, leading to worse compressive strength in the AFP laminates. This is due to excessive compressive pressure from the rigid compaction roller. Another problem inherent to AFP with ISC is the uneven temperature distributions in the through thickness direction of the laminate, causing warpage. The authors recommended heating the mandrel or tool above the $T_{g}$ of the thermoplastic matrix to circumvent the warpage problem. 

Stokes-Griffin and Compston studied various aspects of the AFP process with laser ISC for carbon/PEEK prepreg tapes [52,79]. Laminates fabricated using a lower line speed (100 mm/s) exhibited better fiber-matrix bonding compared to laminates fabricated using a higher line speed (400 mm/s). The authors attributed this to the higher crystallinity in the laminates fabricated at 100 mm/s. For the compaction roller, a deformable roller (silicone roller) resulted in better consolidation compared to a rigid roller (brass roller). This is due to the large laser shadow produced by the rigid roller. For the modeling of the consolidation process, the authors showed that the temperature threshold for autohesion of PEEK to occur is $T_{g}$ instead of $T_{m}$ if the PEEK is completely melted at the heating phase. 

Doan and Mertiny studied the creep response of FRTP components fabricated using FW with hot air blower ISC [108]. Glass/HDPE prepreg tapes with a width of 49 mm were used. Two batches of specimens were fabricated. For batch 1, the heater setting of 400 °C was used for the first 7 layers and 420 °C was used for layers 8 to 10. For batch 2, the heater was set to 400 °C for the first 4 layers, 420 °C for layers 5 to 8, and 440 °C for layers 9 to 10. The batch 2 specimens exhibited better fiber packing and greater resistance to creep. The authors explained that the higher processing temperatures in the batch 2 specimens led to higher crystallinity, thus resulting in improved creep resistance.

Samak et al. studied the ILSS and void content of ring specimens with layup configurations [0/90]_n_, [0/±30]_n_ and [0/±45]_n_ fabricated using FW with laser ISC [109]. Carbon/PEEK prepreg tapes were wound on a mandrel with a diameter of 400 mm. Gaps between the FRTP tapes was programmed into the FW process. For 90° layers, the gap was set to 1.6 mm while for all other layers, the gap was 1.4 mm. The width of the prepreg tape was not reported. The processing parameters, 7 m/min line speed, 430 °C laser temperature and 600 N compaction force, were kept constant across the specimens. The authors reported the [0/±45]_n_ specimens had the highest ILSS (average of 48.2 MPa) and lowest void content. This suggests that the processing parameters need to be optimized for specimens with different layup configurations.

Peeters et al. studied the fabrication of FRTP composite stiffeners using AFP with laser ISC [14]. For the fiber placement at the corners of the stiffeners, a second pass or repass, where heat and compaction pressure are applied to the FRTP layer without adding new material, was required. The repass was performed to ensure that the prepreg tapes could bond well at the corners. Shadmehri et al. also studied the application of repass treatments in the AFP process with hot gas torch ISC [95]. The repass treatment was found to reduce the surface roughness of the resulting FRTP component. However, the crystallinity of the thermoplastic matrix was also reduced. The authors recommended applying the repass treatment for FRTP components used in aerodynamic applications but doing the repass only at the outermost layer. 

Another study on the effect of repass in AFP with laser ISC was conducted by Chanteli et al. [94]. Four repass methods were compared, namely single repass, double repass, perpendicular repass (repass applied perpendicular to the fiber direction) and tool-side repass (repass applied on the ply already laid on the tool). Specimens fabricated using AFP without repass, with and without an additional consolidation step using autoclave, were also tested. The repass treatments improved the physical properties of the composites (lower void content, lower surface roughness, higher crystallinity) compared to AFP specimens without repass. For the mechanical properties, the single repass treatment was found to improve the open hole compression and in-plane shear performances of the specimens, while the double repass treatment resulted in the worst performance. However, specimens consolidated using autoclave showed the best performance for all the properties studied, especially for the mechanical properties. Comparing the findings from [95] and [94], it can be seen that the effect of the repass treatment depends on the heat source used. For laser ISC, due to the high heating and cooling rate, a repass treatment was required to increase the crystallinity.

Zhao et al. studied the effect of the multi-pass layup process on the interlaminar bonding of FRTP laminates fabricated using AFP [110]. Hot gas torch was used for ISC. For the fabrication of laminates with multiple FRTP layers, the applied heat and compaction pressure affects not only the current layer being placed, but also the substrate as well. Therefore, the applied compaction pressure needs to be varied between layers to achieve uniform interlaminar bonding across the different layers. The authors calculated the optimized compaction pressure for each layup pass by analyzing the intimate contact at the interlaminar regions.

Many heat transfer studies have been conducted to investigate the temperature field in the FRTP composite during FW or AFP with ISC. Weiler et al. presented an analytical solution to the transient thermal analysis of AFP with laser ISC [62]. The authors showed that in the cooling stage of laser ISC (after the nip point), the heat transfer between the FRTP tape and the environment is negligible compared to the internal conduction within the tape. Therefore, the tape can be considered insulated in the cooling stage, thus simplifying the analysis. The authors found that for high speed processes with high laser intensity, the pool of molten polymer is small due to rapid cooling. The authors suggested that this can be improved by increasing the heating length. This can be achieved using laser systems with adjustable heating lengths, such as vertical-cavity surface-emitting lasers (VCSELs) and diode lasers with motorized zoom homogenizer.

Obtaining an analytical solution for the heat transfer analysis in ISC require some simplifications to the problem. On the other hand, numerical simulations enable various boundary conditions, heat flux distributions and temperature-dependent material properties to be considered, thus leading to more accurate predictions provided the simulations are set up properly. Kollmannsberger et al. discussed using numerical simulation to obtain the temperature profile at the nip point for AFP with laser ISC [71]. FDM was used to solve the heat equations. For accurate temperature predictions, the authors recommended using values obtained from experiments for the transverse thermal conductivity of the prepreg tape and the thermal contact resistance between the tapes.

Baho et al. studied the effect of the compaction force on the heat flux distribution at the nip point for laser ISC using a ray tracing algorithm [64]. Higher compaction force resulted in larger deformation in the compaction roller. This in turn causes the heated length to be shorter and the heating to be more focused, leading to higher temperature in the material. The increased temperature due to high compaction force need to be taken into consideration to prevent overheating and material degradation.

Cao et al. studied the heat transfer processes in AFP with ISC using hot gas torch for carbon/PEEK by conducting FE simulations and experiments for verification [67]. In particular, the effect of tool temperature on the consolidation of the first layer was investigated. The authors recommended setting the tool temperature to 150 °C, which is slightly above the $T_{g}$ of PEEK, for placing the first layer. Liu et al. also presented a numerical study on the placement of the first layer in AFP with ISC using hot gas torch for carbon/PEEK [66]. The simulated AFP was performed on a convex tool with a radius of curvature of 600 mm. The authors suggested a line speed of 5 mm/s, hot gas temperature of 750 °C and tool temperature of 145 °C for the first layer. The suggested parameters were similar to those recommended by Cao et al. [67]. In addition, Liu et al. also recommended using a Teflon tape to protect the rubber compaction roller for the AFP process [66].

Schlottermuller et al. studied the thermal residual stresses in FRTP components fabricated using FW with ISC [104]. Both simulations and experiments were carried out in the study. The winding angle, the annealing process after winding, the number of layers and mandrel heating were identified as the main factors affecting thermal residual stresses. Dedieu et al. developed FE models to determine the residual stresses in carbon/PEEK rings fabricated using FW with ISC [105]. For the hoop wound rings, residual stresses due to heat were insignificant because the coefficient of thermal expansion (CTE) for carbon/PEEK is small in the fiber direction. The tape tension, the curvature of the tapes during winding and the compaction pressure were the main contributors to residual stresses.

Fricke and Fischer performed the simulations of flat CFRTP panels fabrication using AFP with laser ISC to study the crystallinity and thermal residual stresses [73]. The prepreg tape studied consisted of carbon fibers and low-melt polyaryletherketone (LM-PAEK) as matrix. The crystallization model developed by Gordnian was included in the material model [103]. Crystallization parameters for LM-PAEK were obtained from DSC experiments. Using the process simulation, the researchers found that the unidirectional CFRTP panels exhibited high thermal residual stresses at the interlaminar regions. This caused warpage in the panel, with a calculated maximum deformation of 42 mm.

For the optimization of FW and AFP processes with ISC, due to the large number of variables involved, many researchers used mathematical models and numerical methods such as FE simulations [35,41,66]. Dobrzanski et al. used the Taguchi method in optimizing the processing parameters for FW with ISC [111]. The material system studied was glass/PP commingled tapes. The authors reported optimal nip point temperatures of 230 °C and 210 °C for best tensile strength and best shear performance respectively. This suggests that optimization studies using different performance indicators can lead to different optimized parameters. 

Few studies have been conducted on the fabrication of complex FRTP composite components using AFP with ISC. Clancy et al. studied the fabrication of variable angle tow (VAT) laminates using AFP with laser ISC [94,112]. The authors showed that the AFP process can produce carbon/PEEK VAT laminates with no significant defects for a steering radius of 400 mm and above. However, further studies are required to better understand the effects of AFP processing parameters on the fabrication of FRTP VAT laminates.

Zenker and Gnaedinger studied the application of AFP with laser ISC in making FRTP preforms with variable fiber orientations [113]. Preforms with steered fiber configurations with steering radii in the range of 221 to 1290 mm were laid. Two nip point temperature settings, 220 and 290 °C, were investigated. Carbon/PA6 prepreg tapes with a width of 6.35 mm (or ¼”) were used. The authors reported that the gaps in the preforms grew larger as steering radius decreased below 500 mm. For the lower nip point temperature of 220 °C, the placed tapes exhibited a pattern with local waviness connected by short straight sections. This is due to the lower bond strength in the tape, caused by the lower nip point temperature. For the nip point temperature of 290 °C, continuous waviness in the placed tapes was observed, indicating stronger bonding. 

Tannous et al. studied the effects of FRTP prepreg tape surface friction on the FW process with ISC [114]. The FW process was simulated using FE modeling. The authors showed that friction forces between the prepreg tape, the compaction roller and the mandrel are important in keeping the placed tape in position. Insufficient friction can lead to poor bonding, especially in cases such as components with concave shapes and FW of helical layers. 

Kollmannsberger studied the AFP process with laser ISC for the fabrication of 2D and 3D FRTP composite parts [115]. For 3D parts featuring corners with a small radius, using processing parameters optimized for 2D parts will lead to overheating and degradation of the thermoplastic. This is due to the short laser heating length on the substrate at the corner. Kollmannsberger investigated two control strategies for the laser to prevent overheating, namely predictive closed loop control (PCLC) and coordinate controlled process parameters (CCPP). In PCLC, the laser power is lowered when the start of overheating is detected (using an IR camera). In CCPP, the laser power and the laser angle are controlled based on the position of the AFP head. Both strategies were able to prevent overheating in the fabrication of 3D FRTP composite parts.

A summary of the optimized processing parameters used in past studies is shown in Table 1. Using the AFP and FW processing parameters, researchers were able to fabricate FRTP composite parts with properties better than or comparable to parts fabricated using conventional methods. Many researchers used FRTP prepreg tapes in their studies. For the ISC heat source, the use of high powered lasers enables the AFP and FW processes to be carried out at high line speeds. 

It is noted that the optimization of the processing parameters is dependent on the machine set up and the material system used. Additionally, the optimized processing parameters can vary when optimized based on different performance indicators. Therefore, the parameters shown in Table 1 should be used only as guides in future studies.

## 9. Future Perspectives

Towards the quest for automating the manufacturing process for composite structural parts, both FW and AFP are providing significant benefits in terms of improved production time. However, it is also important to make the processes more flexible to accommodate various design choices. For both processes, there is a growing challenge to meet industrial requirements to accommodate more complex component shapes and material microstructures [118]. For the fabrication of components with complex shapes, the size of the heat source used for ISC needs to be small. Particular attention also needs to be given to the heat transfer and consolidation processes, especially when the component shape involves sharp corners and curves with low radii of curvature [113,115]. Further research on process modeling techniques, including machine learning based methods, can aid in the design and fabrication of composite components with complex shapes [16,119,120].

Besides this, the development of new textile architectures such as thin spread tow fabrics [121,122,123] and the usage of novel resin systems can be coupled to ease the challenges associated with producing high performance composite parts. The development of thin ply fabrics is interesting for many industrial applications due to their potential to significantly improve the in situ mechanical properties and make the composite parts lighter. They have been mostly used in conjunction with liquid injection processes [124,125,126,127,128]. Recently, thin ply thermoplastic prepreg tapes and thin ply dry tapes have been manufactured successfully and made available [129,130,131,132]. However, challenges remain in adapting the FW and AFP processes to use the tapes. Most importantly, the laying mechanisms, the filament wetting and solidification processes are major areas of research which require critical attention. For the processing of thin ply thermoplastic prepreg tapes, the effect of the reduced tape thickness on the heat transfer process during ISC needs to be considered. On the other hand, recently developed liquid resin systems such as the reactively processed thermoplastic Elium from Arkema can be used in conjunction with thin ply dry tapes. Elium is a thermoplastic acrylic based resin in liquid state which can be processed at room temperature [55,124,128,133,134]. It can be used in the FW process with ISC using ultraviolet rays [135]. The development and qualification of the FW process and potentially also the AFP process to effectively use this kind of reactively processed thermoplastic resin systems could be an intriguing research topic.

## 10. Concluding Remarks

The fabrication of FRTP composite components using AFP or FW with ISC is a complex process involving many interconnected factors. Implementing the AFP and FW processes requires careful considerations on the material system and its form, as well as the heat source for ISC. When prepreg tapes are used, the FRTP consolidation process only involves the bonding between the tapes. Hence, it is faster compared to consolidation processes using other semi-finished forms, which require an impregnation step. For the ISC heat source, high powered lasers, with their very high heating rates, enable the AFP and FW processes to be carried out at high line speeds. However, using prepreg tapes and high powered lasers also leads to higher costs. Therefore, there is a need to strike a balance between costs and productivity for the manufacturing process.

Researchers have developed various models to relate the processing parameters to the degree of consolidation and other aspects of FRTP composites. Using the models, along with experimental methods and numerical simulations, many researchers have fabricated good quality FRTP composite components using AFP and FW with ISC. For the quality assessment of the FRTP composite components, many researchers focused on the void content and the interlaminar bonding of the components. However, some researchers have reported components with low void content and good interlaminar bonding showing poor in-plane mechanical properties. Therefore, for a comprehensive assessment, other properties of the FRTP composite components need to be evaluated as well. The processing parameters used in past studies are optimized for specific machine set ups and material systems. Using the reported processing parameters as guidance, the productivity of the AFP and FW processes can be further improved in future studies.

Currently, many studies have focused on composite parts with simple shapes, such as flat plates and tubes with constant radii. For the fabrication of complex parts, additional aspects, such as the heating length at sharp corners, need to be considered in the process controls. More studies on the fabrication of complex FRTP composite components are required to fully realize the potential of the AFP and FW processes. Further research is also needed to expand the application of the manufacturing processes to accommodate the use of innovative material systems, such as thin ply composites and liquid thermoplastic resins.

## Figures and Tables

**Table 1:** Summary of optimized processing parameters from past studies.

Year	Matrix	Material form	Process	Heat Source	Nip Point Temperature (°C)	Line Speed (mm/s)	Compaction Force/Pressure	Other Processing Parameters	Physical and Mechanical Properties Studied	Ref.
2017	PA6	Aramid/PA6 commingled yarns	FW	Hot gas torch	320	10	190 N	Preheating of yarns to 330 °C	Void content, SBS strength, flexural modulus	[36]
2019	PA6	Carbon/PA6 prepreg tapes	AFP	NIR diode laser	260	100	130 N		Wedge peel strength	[53]
2019	PA6	Carbon/PA6 prepreg tapes	FW	Laser	280 (hoop layer), 300 (axial layer)	105 (hoop layer), 50 (axial layer)	0.3 MPa		Void content, compression modulus, implosion strength (for composite tube)	[6]
1993	PA12	Glass/PA12 powder impregnated fibers	FW	Hot gas torch	360	25	157 N	IR preheating of fibers at power 9.6 kW, mandrel temperature 100 °C	Interlaminar shear, fracture toughness (from DCB tests)	[47]
2016	PP	E-glass/PP commingled yarns	FW	Ultrasonic welding	223	52.5	85 N (applied at sonotrode)	Compaction roller located 45 mm from the sonotrode	Void content, shear modulus	[35]
2017	PP	E-glass/PP prepreg tapes	AFP	Ultrasonic welding	260	1	0.15 MPa (applied at compaction roller)	Ultrasonic amplitude 3 µm, frequency 40 kHz	SBS strength, fracture toughness (from DCB tests), impact toughness	[56]
2011	PP	Glass/PP commingled fibers	FW	Hot gas torch	125	42	54 N	Preheating of fibers to 195 °C	Flexural modulus and strength, Charpy impact toughness	[116]
2013	PPS	Carbon/PPS prepreg tapes	AFP	Laser	480	125	450 N	Laser power 1700 W	Fracture toughness (using mandrel peel test)	[41]
1995	PEI	Carbon/PEI powder impregnated fibers	FW	IR spot heater	960	80	Nil	Fiber tension 5 N per 1000 filaments, mandrel temperature 150 °C	Void content	[49]
1996	PEEK	AS4 carbon/PEEK prepreg tapes	FW	CO_2_ laser	475	15	13.8 MPa	Laser power 50 W	SBS strength, fracture toughness (from DCB tests), wedge peel force	[42]
2015	PEEK	AS4 carbon/PEEK prepreg tapes	AFP	NIR diode laser	500	100	500 N	Laser power 920 W	SBS strength	[52]
2019	PEEK	Carbon/PEEK prepreg tapes	AFP	NIR diode laser	485	100	0.34 MPa (for 1st ply), 0.46 MPa (for plies 2–7)	Laser power up to 4 kW	Lap shear strength	[117]

## Data Availability

The data presented in this study are available on request from the corresponding author.

## References

[1] Boon Y.D., Joshi S.C., Ong L.S. (2018). Interfacial bonding between CFRP and mechanically-treated aluminum liner surfaces for risers. Compos. Struct..

[2] Ishikawa T., Amaoka K., Masubuchi Y., Yamamoto T., Yamanaka A., Arai M., Takahashi J. (2018). Overview of automotive structural composites technology developments in Japan. Compos. Sci. Technol..

[3] Bhudolia S.K., Perrotey P., Joshi S.C. (2015). Experimental investigation on suitability of carbon fibre thin plies for racquets. Proc. Inst. Mech. Eng. Part P J. Sports Eng. Technol..

[4] Arhant M., Davies P., Pemberton R., Summerscales J., Graham-Jones J. (2019). 2-Thermoplastic matrix composites for marine applications. Marine Composites.

[5] Mahieux C.A. (2001). Cost effective manufacturing process of thermoplastic matrix composites for the traditional industry: The example of a carbon-fiber reinforced thermoplastic flywheel. Compos. Struct..

[6] Arhant M., Briançon C., Burtin C., Davies P. (2019). Carbon/Polyamide 6 thermoplastic composite cylinders for deep sea applications. Compos. Struct..

[7] Khennane A., Bai J. (2013). 8-Filament winding processes in the manufacture of advanced fibre-reinforced polymer (FRP) composites. Advanced Fibre-Reinforced Polymer (FRP) Composites for Structural Applications.

[8] Beland S. (1990). High Performance Thermoplastic Resins and Their Composites.

[9] Chang I.Y., Lees J.K. (1988). Recent development in thermoplastic composites: A review of matrix systems and processing methods. J. Thermoplast. Compos. Mater..

[10] Fernández I., Blas F., Frövel M. (2003). Autoclave forming of thermoplastic composite parts. J. Mater. Process. Technol..

[11] Sherwood J.A., Fetfatsidis K.A., Gorczyca J.L., Advani S.G., Hsiao K.-T. (2012). 6-Fabric thermostamping in polymer matrix composites. Manufacturing Techniques for Polymer Matrix Composites (PMCs).

[12] Kozaczuk K. (2016). Automated fiber placement systems overview. Trans. Inst. Aviat..

[13] Denkena B., Schmidt C., Weber P. (2016). Automated fiber placement head for manufacturing of innovative aerospace stiffening structures. Procedia Manuf..

[14] Peeters D., Clancy G., Oliveri V., O’Higgins R., Jones D., Weaver P.M. (2019). Concurrent design and manufacture of a thermoplastic composite stiffener. Compos. Struct..

[15] He K., Nie H., Yan C. (2016). The intelligent composite panels manufacturing technology based on tape-laying automatic system. Procedia CIRP.

[16] Sacco C., Baz Radwan A., Anderson A., Harik R., Gregory E. (2020). Machine learning in composites manufacturing: A case study of automated fiber placement inspection. Compos. Struct..

[17] Croft K., Lessard L., Pasini D., Hojjati M., Chen J., Yousefpour A. (2011). Experimental study of the effect of automated fiber placement induced defects on performance of composite laminates. Compos. Part A Appl. Sci. Manuf..

[18] Nguyen M.H., Vijayachandran A.A., Davidson P., Call D., Lee D., Waas A.M. (2019). Effect of automated fiber placement (AFP) manufacturing signature on mechanical performance of composite structures. Compos. Struct..

[19] Zhang P., Sun R., Zhao X., Hu L. (2015). Placement suitability criteria of composite tape for mould surface in automated tape placement. Chin. J. Aeronaut..

[20] Oromiehie E., Prusty B.G., Compston P., Rajan G. (2019). Automated fibre placement based composite structures: Review on the defects, impacts and inspections techniques. Compos. Struct..

[21] Dhinakaran V., Surendar K.V., Hasunfur Riyaz M.S., Ravichandran M. (2020). Review on study of thermosetting and thermoplastic materials in the automated fiber placement process. Mater. Today Proc..

[22] Gonzalez Henriquez R., Mertiny P., Beaumont P.W.R., Zweben C.H. (2018). 3.21 Filament winding applications. Comprehensive Composite Materials II.

[23] Bhudolia S., Fischer S., He P., Yue C.Y., Joshi S.C., Yang J. (2015). Design, manufacturing and testing of filament wound composite risers for marine and offshore applications. Mater. Sci. Forum.

[24] Sorrentino L., Anamateros E., Bellini C., Carrino L., Corcione G., Leone A., Paris G. (2019). Robotic filament winding: An innovative technology to manufacture complex shape structural parts. Compos. Struct..

[25] Beck B., Tawfik H., Haas J., Park Y.B., Henning F. (2020). Automated 3D skeleton winding process for continuous-fiber-reinforcements in structural thermoplastic components. Advances in Polymer Processing 2020, Proceedings of the International Symposium on Plastics Technology, Aachen, Germany, 10 March 2020.

[26] Wiegand N., Mäder E. (2017). Commingled yarn spinning for thermoplastic/glass fiber composites. Fibers.

[27] Boon Y.D., Joshi S.C. (2020). A review of methods for improving interlaminar interfaces and fracture toughness of laminated composites. Mater. Today Commun..

[28] Gabrion X., Placet V., Trivaudey F., Boubakar L. (2016). About the thermomechanical behaviour of a carbon fibre reinforced high-temperature thermoplastic composite. Compos. Part B Eng..

[29] Mack J., Schledjewski R., Advani S.G., Hsiao K.-T. (2012). 7-Filament winding process in thermoplastics. Manufacturing Techniques for Polymer Matrix Composites (PMCs).

[30] Volk M., Wong J., Arreguin S., Bar C., Schmuck F., Ermanni P. Thermoplastic composite materials for high voltage insulator applications. Proceedings of the ECCM18—18th European Conference on Composite Materials.

[31] Iannone V., Barile M., Lecce L. (2018). Automated fabrication of hybrid thermoplastic prepreg material to be processed by in-situ consolidation automated fiber placement process. MATEC Web Conf..

[32] Friedrich K., Karger-Kocsis J. (1999). Commingled yarns and their use for composites. Polypropylene: An A–Z Reference.

[33] Bernet N., Michaud V., Bourban P.E., Månson J.A.E. (2001). Commingled yarn composites for rapid processing of complex shapes. Compos. Part A Appl. Sci. Manuf..

[34] Thomann U.I., Ermanni P. (2004). The influence of yarn structure and processing conditions on the laminate quality of stampformed carbon and thermoplastic polymer fiber commingled yarns. J. Thermoplast. Compos. Mater..

[35] Lionetto F., Dell’Anna R., Montagna F., Maffezzoli A. (2016). Modeling of continuous ultrasonic impregnation and consolidation of thermoplastic matrix composites. Compos. Part A Appl. Sci. Manuf..

[36] Wong J.C.H., Blanco J.M., Ermanni P. (2017). Filament winding of aramid/PA6 commingled yarns with in situ consolidation. J. Thermoplast. Compos. Mater..

[37] Vodermayer A.M., Kaerger J.C., Hinrichsen G. (1993). Manufacture of high performance fibre-reinforced thermoplastics by aqueous powder impregnation. Compos. Manuf..

[38] Goud V., Alagirusamy R., Das A., Kalyanasundaram D. (2018). Dry electrostatic spray coated towpregs for thermoplastic composites. Fibers Polym..

[39] Henninger F., Friedrich K. (2002). Thermoplastic filament winding with online-impregnation. Part A: Process technology and operating efficiency. Compos. Part A Appl. Sci. Manuf..

[40] Henninger F., Hoffmann J., Friedrich K. (2002). Thermoplastic filament winding with online-impregnation. Part B. Experimental study of processing parameters. Compos. Part A Appl. Sci. Manuf..

[41] Grouve W.J.B., Warnet L.L., Rietman B., Visser H.A., Akkerman R. (2013). Optimization of the tape placement process parameters for carbon–PPS composites. Compos. Part A Appl. Sci. Manuf..

[42] Agarwal V., McCullough R.L., Schultz J.M. (1996). The thermoplastic laser-assisted consolidation process-mechanical and microstructure characterization. J. Thermoplast. Compos. Mater..

[43] Funck R., Neitzel M. (1995). Improved thermoplastic tape winding using laser or direct-flame heating. Compos. Manuf..

[44] Yassin K., Hojjati M. (2018). Processing of thermoplastic matrix composites through automated fiber placement and tape laying methods:A review. J. Thermoplast. Compos. Mater..

[45] Tafreshi O.A., Hoa S.V., Shadmehri F., Hoang D.M., Rosca D. (2020). Determination of convective heat transfer coefficient for automated fiber placement (AFP) for thermoplastic composites using hot gas torch. Adv. Manuf. Polym. Compos. Sci..

[46] Rizzolo R.H., Walczyk D.F. (2015). Ultrasonic consolidation of thermoplastic composite prepreg for automated fiber placement. J. Thermoplast. Compos. Mater..

[47] Lauke B., Friedrich K. (1993). Evaluation of processing parameters of thermoplastic composites fabricated by filament winding. Compos. Manuf..

[48] Hoa S.V., Hoang M.D., Simpson J. (2017). Manufacturing procedure to make flat thermoplastic composite laminates by automated fibre placement and their mechanical properties. J. Thermoplast. Compos. Mater..

[49] Romagna J., Ziegmann G., Flemming M. (1995). Thermoplastic filament winding—An experimental investigation of the on-line consolidation of poly(ether imide) fit preforms. Compos. Manuf..

[50] Deden D., Bruckner F., Brandt L., Fischer F. Comparison of heat sources for automated dry fibre placement: Xenon flashlamp vs. infrared heating. Proceedings of the 22nd International Conference on Composites Materials ICCM22.

[51] Comer A.J., Ray D., Obande W.O., Jones D., Lyons J., Rosca I., O’ Higgins R.M., McCarthy M.A. (2015). Mechanical characterisation of carbon fibre—PEEK manufactured by laser-assisted automated-tape-placement and autoclave. Compos. Part A Appl. Sci. Manuf..

[52] Stokes-Griffin C.M., Compston P. (2015). The effect of processing temperature and placement rate on the short beam strength of carbon fibre–PEEK manufactured using a laser tape placement process. Compos. Part A Appl. Sci. Manuf..

[53] Stokes-Griffin C.M., Kollmannsberger A., Compston P., Drechsler K. (2019). The effect of processing temperature on wedge peel strength of CF/PA6 laminates manufactured in a laser tape placement process. Compos. Part A Appl. Sci. Manuf..

[54] Stokes-Griffin C.M., Compston P. (2015). A combined optical-thermal model for near-infrared laser heating of thermoplastic composites in an automated tape placement process. Compos. Part A Appl. Sci. Manuf..

[55] Bhudolia S.K., Gohel G., Leong K.F., Islam A. (2020). Advances in ultrasonic welding of thermoplastic composites: A review. Materials.

[56] Chu Q., Li Y., Xiao J., Huan D., Zhang X., Chen X. (2017). Processing and characterization of the thermoplastic composites manufactured by ultrasonic vibration-assisted automated fiber placement. J. Thermoplast. Compos. Mater..

[57] Dell’Anna R., Lionetto F., Montagna F., Maffezzoli A. (2018). Lay-Up and consolidation of a composite pipe by in situ ultrasonic welding of a thermoplastic matrix composite tape. Materials.

[58] Heraeus Noblelight humm3®—Intelligent Heat for Automated Fibre Placement (AFP). https://www.heraeus.com/en/hng/products_and_solutions/arc_and_flash_lamps/humm3/humm3.html.

[59] Nguyen C., Kolbe A., Bans C. Application of lightning strike protection on thermoplastic structures by automated fiber placement. Proceedings of the 5th International Conference and Exhibition on Thermoplastic Composites ITHEC.

[60] Whitaker S. (1977). Fundamental Principles of Heat Transfer.

[61] Sonmez F.O., Hahn H.T. (1997). Modeling of heat transfer and crystallization in thermoplastic composite tape placement process. J. Thermoplast. Compos. Mater..

[62] Weiler T., Emonts M., Wollenburg L., Janssen H. (2017). Transient thermal analysis of laser-assisted thermoplastic tape placement at high process speeds by use of analytical solutions. J. Thermoplast. Compos. Mater..

[63] Stokes-Griffin C.M., Compston P., Matuszyk T.I., Cardew-Hall M.J. (2013). Thermal modelling of the laser-assisted thermoplastic tape placement process. J. Thermoplast. Compos. Mater..

[64] Baho O., Ausias G., Grohens Y., Férec J. (2020). Simulation of laser heating distribution for a thermoplastic composite: Effects of AFP head parameters. Int. J. Adv. Manuf. Technol..

[65] Venkateshan S.P. (2021). Heat Transfer.

[66] Liu X.L., Han Z.Y., Zhao Z.Y., Sun S.Z. (2021). Thermal analysis of cylindrical molds using thermoplastic composite during robotic fiber placement. J. Phys. Conf. Ser..

[67] Cao Z., Dong M., Liu K., Fu H. (2020). Temperature field in the heat transfer process of PEEK thermoplastic composite fiber placement. Materials.

[68] Tafreshi O.A., Hoa S.V., Shadmehri F., Hoang D.M., Rosca D. (2019). Heat transfer analysis of automated fiber placement of thermoplastic composites using a hot gas torch. Adv. Manuf. Polym. Compos. Sci..

[69] Zacherl L., Shadmehri F., Rother K. (2021). Determination of convective heat transfer coefficient for hot gas torch (HGT)-assisted automated fiber placement (AFP) for thermoplastic composites. J. Thermoplast. Compos. Mater..

[70] Agarwal V., Guçeri S.I., McCullough R.L., Schultz J.M. (1992). Thermal characterization of the laser-assisted consolidation process. J. Thermoplast. Compos. Mater..

[71] Kollmannsberger A., Lichtinger R., Hohenester F., Ebel C., Drechsler K. (2017). Numerical analysis of the temperature profile during the laser-assisted automated fiber placement of CFRP tapes with thermoplastic matrix. J. Thermoplast. Compos. Mater..

[72] Song X. (2000). Modeling of Thermoplastic Composite Filament Winding. Master’s Thesis.

[73] Fricke D., Fischer F. Process simulation of the in-situ automated fiber placement process for thermoplastic composites. Proceedings of the SAMPE Europe Conference.

[74] Dara P.H., Loos A.C. (1985). Thermoplastic Matrix Composite Processing Model.

[75] Loos A.C., Dara P.H. (1987). Processing of thermoplastic matrix composites. Review of Progress in Quantitative Nondestructive Evaluation.

[76] Lee W.I., Springer G.S. (1987). A model of the manufacturing process of thermoplastic matrix composites. J. Compos. Mater..

[77] Mantell S.C., Springer G.S. (1992). Manufacturing process models for thermoplastic composites. J. Compos. Mater..

[78] Yang F., Pitchumani R. (2001). A fractal Cantor set based description of interlaminar contact evolution during thermoplastic composites processing. J. Mater. Sci..

[79] Stokes-Griffin C.M., Compston P. (2016). Investigation of sub-melt temperature bonding of carbon-fibre/PEEK in an automated laser tape placement process. Compos. Part A Appl. Sci. Manuf..

[80] Yang F., Pitchumani R. (2002). Healing of thermoplastic polymers at an interface under nonisothermal conditions. Macromolecules.

[81] Boiko Y.M., Guérin G., Marikhin V.A., Prud’homme R.E. (2001). Healing of interfaces of amorphous and semi-crystalline poly(ethylene terephthalate) in the vicinity of the glass transition temperature. Polymer.

[82] De Gennes P.G. (1971). Reptation of a polymer chain in the presence of fixed obstacles. J. Chem. Phys..

[83] Kim Y.H., Wool R.P. (1983). A theory of healing at a polymer-polymer interface. Macromolecules.

[84] Wool R.P., Yuan B.-L., McGarel O.J. (1989). Welding of polymer interfaces. Polym. Eng. Sci..

[85] Yang F., Pitchumani R. (2003). Nonisothermal healing and interlaminar bond strength evolution during thermoplastic matrix composites processing. Polym. Compos..

[86] Bernet N., Michaud V., Bourban P.E., Manson J.A.E. (1999). An impregnation model for the consolidation of thermoplastic composites made from commingled yarns. J. Compos. Mater..

[87] Klinkmüller V., Um M.K., Steffens M., Friedrich K., Kim B.S. (1994). A new model for impregnation mechanisms in different GF/PP commingled yarns. Appl. Compos. Mater..

[88] Gutowski T.G., Cai Z., Bauer S., Boucher D., Kingery J., Wineman S. (1987). Consolidation experiments for laminate composites. J. Compos. Mater..

[89] Ye L., Friedrich K., Kästel J. (1994). Consolidation of GF/PP commingled yarn composites. Appl. Compos. Mater..

[90] Gebart B.R. (1992). Permeability of unidirectional reinforcements for RTM. J. Compos. Mater..

[91] Ye L., Klinkmuller V., Friedrich K. (1992). Impregnation and consolidation in composites made of GF/PP powder impregnated bundles. J. Thermoplast. Compos. Mater..

[92] Steggall-Murphy C., Simacek P., Advani S.G., Yarlagadda S., Walsh S. (2010). A model for thermoplastic melt impregnation of fiber bundles during consolidation of powder-impregnated continuous fiber composites. Compos. Part A Appl. Sci. Manuf..

[93] Connor M., Toll S., Månson J.A.E., Gibson A.G. (1995). A Model for the consolidation of aligned thermoplastic powder impregnated composites. J. Thermoplast. Compos. Mater..

[94] Chanteli A., Bandaru A.K., Peeters D., O’Higgins R.M., Weaver P.M. (2020). Influence of repass treatment on carbon fibre-reinforced PEEK composites manufactured using laser-assisted automatic tape placement. Compos. Struct..

[95] Shadmehri F., Hoa S.V., Fortin-Simpson J., Ghayoor H. (2018). Effect of in situ treatment on the quality of flat thermoplastic composite plates made by automated fiber placement (AFP). Adv. Manuf. Polym. Compos. Sci..

[96] Ozawa T. (1971). Kinetics of non-isothermal crystallization. Polymer.

[97] Joshi S.C., Lam Y.C. (2006). Integrated approach for modelling cure and crystallization kinetics of different polymers in 3D pultrusion simulation. J. Mater. Process. Technol..

[98] Choe C.R., Lee K.H. (1989). Nonisothermal crystallization kinetics of poly(etheretherketone) (PEEK). Polym. Eng. Sci..

[99] Tobin M.C. (1974). Theory of phase transition kinetics with growth site impingement. I. Homogeneous nucleation. J. Polym. Sci. Polym. Phys. Ed..

[100] Tobin M.C. (1976). The theory of phase transition kinetics with growth site impingement. II. Heterogeneous nucleation. J. Polym. Sci. Polym. Phys. Ed..

[101] Tobin M.C. (1977). Theory of phase transition kinetics with growth site impingement. III. Mixed heterogeneous-homogeneous nucleation and nonintegral exponents of the time. J. Polym. Sci. Polym. Phys. Ed..

[102] Maffezzoli A.M., Kenny J.M., Nicolais L. (1989). Welding of PEEK/carbon fiber composite laminates. SAMPE J..

[103] Gordnian K. (2017). Crystallization and Thermo-Viscoelastic Modelling of Polymer Composites. Ph.D. Thesis.

[104] Schlottermuller M., Lu H., Roth Y., Himmel N., Schledjewski R., Mitschang P. (2003). Thermal residual stress simulation in thermoplastic filament winding process. J. Thermoplast. Compos. Mater..

[105] Dedieu C., Barasinski A., Chinesta F., Dupillier J.-M. (2017). About the origins of residual stresses in in situ consolidated thermoplastic composite rings. Int. J. Mater. Form..

[106] Nam J.-D., Seferis J.C. (1992). Generalized composite degradation kinetics for polymeric systems under isothermal and nonisothermal conditions. J. Polym. Sci. Part B Polym. Phys..

[107] Sonmez F.O., Hahn H.T. (1997). Analysis of the on-line consolidation process in thermoplastic composite tape placement. J. Thermoplast. Compos. Mater..

[108] Doan H.G.M., Mertiny P. (2020). Creep testing of thermoplastic fiber-reinforced polymer composite tubular coupons. Materials.

[109] Samak S., Risteska S., Dukovski V., Trajkoski S. (2020). Some experimental investigation of products from thermoplastic composite materials manufactured with robot and LAFP. Int. J. Eng. Res. Technol..

[110] Zhao P., Shirinzadeh B., Shi Y., Cheuk S., Clark L. (2018). Multi-Pass layup process for thermoplastic composites using robotic fiber placement. Robot. Comput. Integr. Manuf..

[111] Dobrzanski L.A., Domagala J., Silva J.F. (2007). Application of Taguchi method in the optimisation of filament winding of thermoplastic composites. Arch. Mater. Sci. Eng..

[112] Clancy G., Peeters D., Oliveri V., Jones D., O’Higgins R.M., Weaver P.M. (2019). A study of the influence of processing parameters on steering of carbon Fibre/PEEK tapes using laser-assisted tape placement. Compos. Part B Eng..

[113] Zenker T., Gnaedinger M. Consolidation behavior of fiber steered thermoplastic automated fiber placement preforms. Proceedings of the 5th International Conference and Exhibition on Thermoplastic Composites ITHEC.

[114] Tannous M., Barasinski A., Binetruy C., Courtemanche B. (2016). Contribution of thermo-mechanical parameters and friction to the bonding of thermoplastic tapes in the tape winding process. J. Mater. Process. Technol..

[115] Kollmannsberger A. (2019). Heating Characteristics of Fixed Focus Laser Assisted Thermoplastic-Automated Fiber Placement of 2D and 3D Parts. Ph.D. Thesis.

[116] Gennaro R., Montagna F., Maffezzoli A., Fracasso F., Fracasso S. (2011). On-Line consolidation of commingled polypropylene/glass roving during filament winding. J. Thermoplast. Compos. Mater..

[117] Engelhardt R., Ehard S., Wolf T., Oelhafen J., Kollmannsberger A., Drechsler K. (2019). In situ joining of unidirectional tapes on long fiber reinforced thermoplastic structures by thermoplastic automated fiber placement for scientific sounding rocket applications. Procedia CIRP.

[118] Frketic J., Dickens T., Ramakrishnan S. (2017). Automated manufacturing and processing of fiber-reinforced polymer (FRP) composites: An additive review of contemporary and modern techniques for advanced materials manufacturing. Addit. Manuf..

[119] Boon Y.D., Joshi S.C., Bhudolia S.K., Gohel G. (2020). Recent advances on the design automation for performance-optimized fiber reinforced polymer composite components. J. Compos. Sci..

[120] Sun S., Han Z., Zhang J., Jin H., Wang Y. (2021). Multiscale collaborative process optimization method for automated fiber placement. Compos. Struct..

[121] Bhudolia S.K., Kam K.K.C., Perrotey P., Joshi S.C. (2018). Effect of fixation stitches on out-of-plane response of textile non-crimp fabric composites. J. Ind. Text..

[122] Bhudolia S.K., Joshi S.C., Bert A., Gohel G.R., Raama M. (2019). Energy characteristics and failure mechanisms for textile spread tow thin ply thermoplastic composites under low-velocity impact. Fibers Polym..

[123] Cugnoni J., Amacher R., Kohler S., Brunner J., Kramer E., Dransfeld C., Smith W., Scobbie K., Sorensen L., Botsis J. (2018). Towards aerospace grade thin-ply composites: Effect of ply thickness, fibre, matrix and interlayer toughening on strength and damage tolerance. Compos. Sci. Technol..

[124] Bhudolia S.K., Gohel G., Leong K.F., Joshi S.C. (2020). Damping, impact and flexural performance of novel carbon/Elium® thermoplastic tubular composites. Compos. Part B Eng..

[125] Bhudolia S., Joshi S., Bert A., Boon Y.D., Makam R., Gohel G. (2019). Flexural characteristics of novel carbon methylmethacrylate composites. Compos. Commun..

[126] Bhudolia S.K., Joshi S.C. (2018). Low-Velocity impact response of carbon fibre composites with novel liquid Methylmethacrylate thermoplastic matrix. Compos. Struct..

[127] Bhudolia S.K., Perrotey P., Joshi S.C. (2018). Mode I fracture toughness and fractographic investigation of carbon fibre composites with liquid Methylmethacrylate thermoplastic matrix. Compos. Part B Eng..

[128] Bhudolia S.K., Gohel G., Joshi S.C., Leong K.F. (2020). Quasi-Static indentation response of core-shell particle reinforced novel NCCF/Elium^®^ composites at different feed rates. Compos. Commun..

[129] Ricard T. (2017). Automated preform manufacture at an affordable price. Reinf. Plast..

[130] Casanovas J., Costa J., Mayugo J.A., Llongueras A. (2018). Fabrication of hybrid thin ply tapes. IOP Conf. Ser. Mater. Sci. Eng..

[131] Galos J. (2020). Thin-Ply composite laminates: A review. Compos. Struct..

[132] Yamashita S., Hirano Y., Sonehara T., Takahashi J., Kawabe K., Murakami T. (2017). Residual mechanical properties of carbon fibre reinforced thermoplastics with thin-ply prepreg after simulated lightning strike. Compos. Part A Appl. Sci. Manuf..

[133] Kazemi M.E., Shanmugam L., Li Z., Ma R., Yang L., Yang J. (2020). Low-Velocity impact behaviors of a fully thermoplastic composite laminate fabricated with an innovative acrylic resin. Compos. Struct..

[134] Bhudolia S.K., Gohel G., Kah Fai L., Barsotti R.J. (2020). Fatigue response of ultrasonically welded carbon/Elium® thermoplastic composites. Mater. Lett..

[135] Arkema Elium Resin: A Disruptive Innovation in the World of Composites?. https://www.arkema.com/global/en/webzine/post/elium-resin-a-disruptive-innovation-in-the-world-of-composites/.

